# Clinical accuracy of instrument-based SARS-CoV-2 antigen diagnostic tests: a systematic review and meta-analysis

**DOI:** 10.1186/s12985-024-02371-5

**Published:** 2024-04-29

**Authors:** Katharina Manten, Stephan Katzenschlager, Lukas E. Brümmer, Stephani Schmitz, Mary Gaeddert, Christian Erdmann, Maurizio Grilli, Nira R. Pollock, Aurélien Macé, Berra Erkosar, Sergio Carmona, Stefano Ongarello, Cheryl C. Johnson, Jilian A. Sacks, Verena Faehling, Linus Bornemann, Markus A. Weigand, Claudia M. Denkinger, Seda Yerlikaya

**Affiliations:** 1 Department of Infectious Disease and Tropical Medicine, Heidelberg University Hospital, Im Neuenheimer Feld 324, 69120 Heidelberg, Germany; 2 Department of Anesthesiology, Heidelberg University Hospital, Heidelberg, Germany; 3 Department of Developmental Biology, Erasmus Medical Center, Rotterdam, Netherlands; 4 FH Muenster University of Applied Sciences, Muenster, Germany; 5 Library, University Medical Center Mannheim, Mannheim, Germany; 6 Department of Laboratory Medicine, Boston Children’s Hospital, Boston, MA USA; 7 FIND, Geneva, Switzerland; 8 Global HIV, Hepatitis and STIs Programmes, World Health Organization, Geneva, Switzerland; 9 Department of Epidemic and Pandemic Preparedness and Prevention, World Health Organization, Geneva, Switzerland; 10 Institute of Virology, Faculty of Medicine, University Medical Centre, University of Freiburg, Freiburg, Germany; 11 German Center for Infection Research (DZIF), partner site Heidelberg University Hospital, Heidelberg, Germany

**Keywords:** COVID-19, Instrument-based, Antigen test, Systematic review, Meta-analysis, SARS-CoV-2

## Abstract

**Background:**

During the COVID-19 pandemic, antigen diagnostic tests were frequently used for screening, triage, and diagnosis. Novel instrument-based antigen tests (iAg tests) hold the promise of outperforming their instrument-free, visually-read counterparts. Here, we provide a systematic review and meta-analysis of the SARS-CoV-2 iAg tests’ clinical accuracy.

**Methods:**

We systematically searched MEDLINE (via PubMed), Web of Science, medRxiv, and bioRxiv for articles published before November 7th, 2022, evaluating the accuracy of iAg tests for SARS-CoV-2 detection. We performed a random effects meta-analysis to estimate sensitivity and specificity and used the QUADAS-2 tool to assess study quality and risk of bias. Sub-group analysis was conducted based on Ct value range, IFU-conformity, age, symptom presence and duration, and the variant of concern.

**Results:**

We screened the titles and abstracts of 20,431 articles and included 114 publications that fulfilled the inclusion criteria. Additionally, we incorporated three articles sourced from the FIND website, totaling 117 studies encompassing 95,181 individuals, which evaluated the clinical accuracy of 24 commercial COVID-19 iAg tests. The studies varied in risk of bias but showed high applicability. Of 24 iAg tests from 99 studies assessed in the meta-analysis, the pooled sensitivity and specificity compared to molecular testing of a paired NP swab sample were 76.7% (95% CI 73.5 to 79.7) and 98.4% (95% CI 98.0 to 98.7), respectively. Higher sensitivity was noted in individuals with high viral load (99.6% [95% CI 96.8 to 100] at Ct-level ≤ 20) and within the first week of symptom onset (84.6% [95% CI 78.2 to 89.3]), but did not differ between tests conducted as per manufacturer’s instructions and those conducted differently, or between point-of-care and lab-based testing.

**Conclusion:**

Overall, iAg tests have a high pooled specificity but a moderate pooled sensitivity, according to our analysis. The pooled sensitivity increases with lower Ct-values (a proxy for viral load), or within the first week of symptom onset, enabling reliable identification of most COVID-19 cases and highlighting the importance of context in test selection. The study underscores the need for careful evaluation considering performance variations and operational features of iAg tests.

**Supplementary Information:**

The online version contains supplementary material available at 10.1186/s12985-024-02371-5.

## Background and rationale

Antigen diagnostic tests have been a key component of the COVID-19 response [1], as they allow early and prompt identification of SARS-CoV-2-positive individuals, especially when the viral load is high [2–6]. Antigen diagnostic tests are immunoassays designed to detect SARS-CoV-2 antigen targets, primarily the nucleocapsid protein. A wide variety of antigen diagnostic tests have been commercialized during the pandemic, enabling use in different settings from self-testing to specialized laboratories [7, 8]. Among those, the instrument-free lateral flow assays (LFAs) are the most widely used antigen detection tests, especially in circumstances where fast results are needed and only limited resources are available (including a lack of highly skilled personnel or specialized laboratory equipment). Their clinical performance has been extensively reviewed and falls short in comparison to the gold standard, reverse transcription-polymerase chain reaction (RT-PCR) [9–11]. On the other hand, instrument-based antigen diagnostic tests (iAg tests) leverage a variety of antigen-detection technologies with varying levels of automation and infrastructure requirements. For instance, iAg tests include high-throughput, fully automated, laboratory-based instruments as well as LFAs with results amplification (e.g., fluorescence) and dedicated readers that enable standardized result interpretation and connectivity. Their diversity encompasses a broad range of potential benefits for streamlining clinical procedures. For instance, some enable the concurrent detection of multiple pathogens [12] and/or expedite and automate testing processes. Consequently, these methods have garnered significant attention, not only for their aforementioned advantages, but also due to their reported higher sensitivity compared to conventional lateral flow tests [9, 13, 14].

There have been a number of systematic evidence syntheses on the diagnostic performance of instrument-free antigen detection tests for SARS-CoV-2 [9, 11, 14–16]; however, the performance of iAg tests has received relatively little attention. Previous review articles combined data from instrument-free and instrument-based antigen tests regardless of their development stage [10, 14]. Only digital and fluorescence immunoassays, which are often more appropriate for point-of-care (POC) use, were considered in the review by Keskin et al., specifically focused on iAg tests [17]. Here, we conducted a systematic review and meta-analysis to assess the clinical performance of commercially available iAg tests for detecting current SARS-CoV-2 infection compared to RT-qPCR and/or culture as the reference standard.

## Methods

### Overview

This study expands on systematic reviews previously published by our group, which assessed the clinical accuracy of commercially available instrument-free and instrument-based antigen-detection rapid diagnostic tests (Ag-RDTs) for SARS-CoV-2 [9, 18, 19]. The methodology of our most recent analysis was applied in the present review as appropriate [19], following the standards of the Preferred Reporting Items for Systematic Reviews and Meta-Analyses (PRISMA) checklist [20] (Supplementary Material, File S1). The study protocol was registered on PROSPERO (ID CRD42021276232) [21].

The following is a summary of the study protocol deviations: Analytical studies were excluded because it was not possible to conduct the analysis due to inconsistent and inadequate reporting and disparate methodologies. For similar reasons, we were unable to include in the analysis all the protocol-specified variables—viral load and antigen levels, for example—that could affect test performance.

### Searched databases

We systematically searched MEDLINE (PubMed), Web of Science, medRxiv, and bioRxiv using search terms and strategy on the basis of the earlier reviews cited above [9, 18]. The full list of search terms was adapted from our previous reviews and is available in Supplementary Material (File S1) [9, 19]. No language or geographic restrictions were applied. Searches were carried out until November 7th, 2022. Additional relevant papers were identified by manually searching the website of FIND, the global alliance for diagnostics, on February 19th, 2023 (https://www.finddx.org/sarscov2-eval-antigen/).

### Eligibility criteria

Clinical studies evaluating the clinical accuracy of commercially available iAg tests for SARS-CoV-2 detection against RT-PCR or viral culture as a reference standard were considered eligible. Retrospective and prospective clinical diagnostic accuracy studies with a cohort, case-control, cross-sectional, or randomized design were eligible. We included both peer-reviewed publications and preprints to present the most recent data. We excluded studies on monitoring or analytical studies and studies with a sample size of less than ten, as smaller sample sizes are prone to yielding erroneous estimations of accuracy.

### Index test

Fully automated, high-throughput laboratory-based iAg tests as well as POC iAg devices requiring a specialized reader (and if the use of the appropriate reader was stated) were included in the review.

In order to further categorize the technologies under investigation, we divided the diverse group of iAg tests based on whether they were applicable to POC (henceforth called ‘POC’) or laboratory-based testing (henceforth called ‘lab-based’). A detailed description of the included tests is provided in Table S1 and File S2.

### Assessment of methodological quality

The QUADAS-2 tool, which had been adjusted for a prior review [9], was used to evaluate the quality of clinical accuracy studies. The relevant publication has comprehensive details on the adjustments that were made [9]. To assess whether studies performed iAg tests as per manufacturers’ instructions for use (IFU), given that it was not possible to trace back version differences across studies due to software and hardware changes, we exclusively relied on the unambiguous statement of IFU compliance, as described in each study.

### Study selection and data extraction

The selection of studies, data extraction, and quality assessment were carried out as described previously [9, 19]. When needed, we reached out to the study authors to request any missing information or clarification. The final data set is available in the Supplementary Material (File S2).

### Statistical analysis and data synthesis

Statistical analysis and data synthesis were conducted as previously established [19]. In summary, we calculated the performance estimates when these outcomes were assessed in at least four studies (with ≥ 20 positive samples). If there were a minimum of four data sets, each including at least 20 RT-PCR-positive samples, a bivariate meta-analysis or a univariate random effects inverse variance meta-analysis was conducted. Otherwise, only a descriptive analysis was carried out. We predefined subgroups for meta-analysis based on the following criteria: Ct value range, testing in accordance with IFU, age, presence of symptoms, duration of symptoms, and viral variant of concern. R 4.2.1 (R Foundation for Statistical Computing, Vienna, Austria) was used for the analyses.

We used the Deeks test for funnel-plot asymmetry to investigate small study effects (the “midas” command in Stata, version 15) [22, 23]. A significant asymmetry is indicated by a p-value for the slope coefficient less than 0.10.

For more information on the methodology, please refer to the original publications [9, 18, 19].

## Results

### Summary of studies

**Fig. 1:**
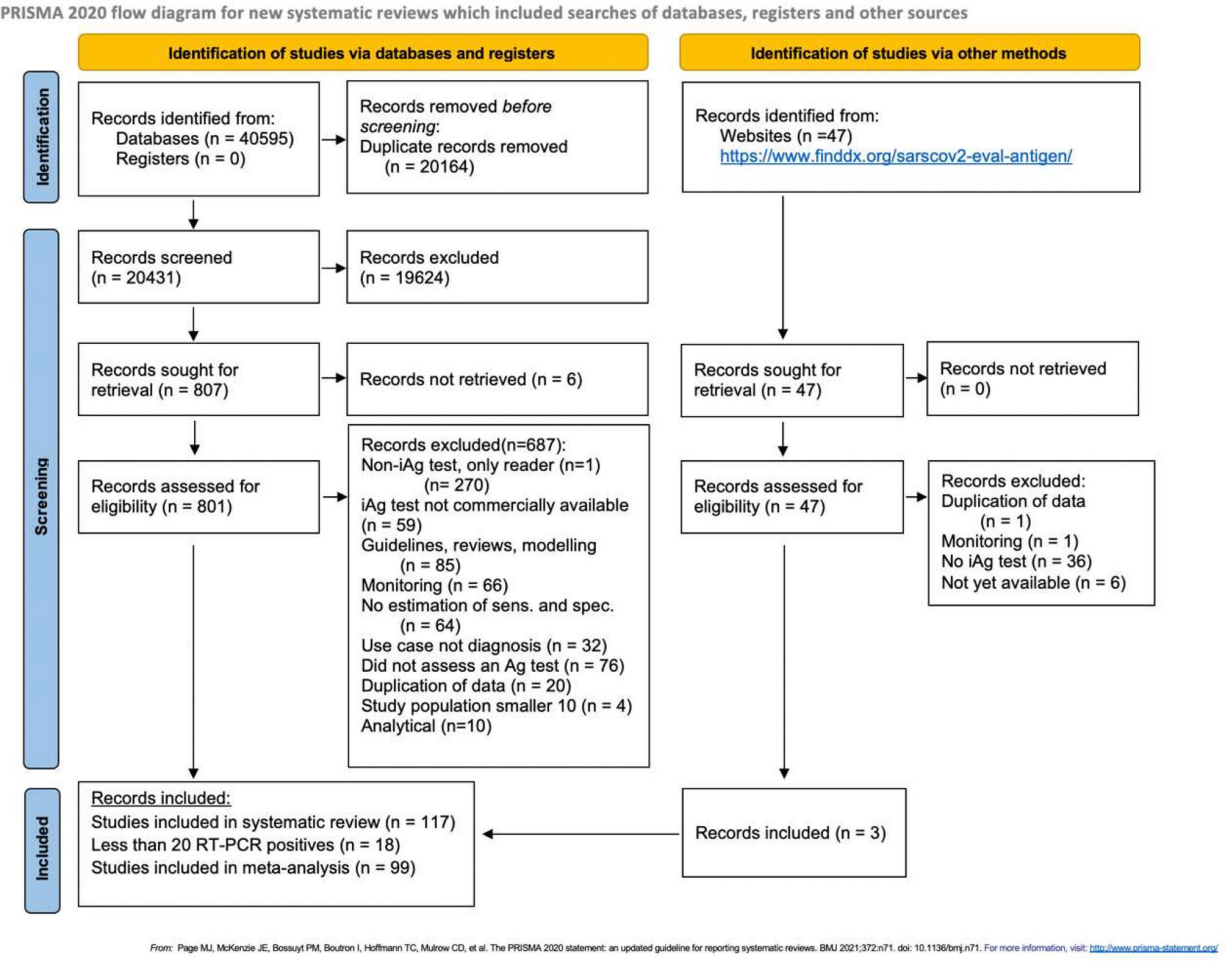
PRISMA flow diagram [20]. *Abbreviations* Ag-RDT = antigen rapid diagnostic test; RT-PCR = reverse transcription polymerase chain reaction; sens = sensitivity; spec = specificity; iAg test = instrument-based antigen test

The systematic search resulted in 40,595 records. After removing duplicates, 20,431 articles were left for title and abstract screening. Out of these, 807 were considered eligible for full-text screening, of which a total of 114 were finally included. Three additional articles were included after screening the articles found on the FIND website. Thus, a total of 117 articles incorporating 159 data sets reporting on 24 unique iAg tests were included in the review (Fig. 1).

### Study description

A total of 109 of the 117 studies included in the review were conducted in high-income countries (HICs) and only seven studies were conducted in low- and middle-income countries (LMICs) [24–30]. Two studies were multi-country studies conducted in the USA/India and the UK/USA [31, 32].

A case-control design was used in 32 of the studies (27.4%) [33–64], while the remaining 85 (72.6%) were cohort studies. RT-PCR was the reference method for all but one study that used viral culture [65]. Out of a total of 159 data sets, 44 (27.7%) reported on adult populations, seven data sets (4.4%) on children, and 32 data sets (20.1%) on mixed populations. In less than half of the data sets (*n* = 76; 47.8%), the age group of the target population was not reported. Across all the studies, the main reasons for testing were screening regardless of symptom status (70/159 data sets, 44.0%), contact investigations (67/159 data sets, 42.1%), and/or presence of symptoms (117/159 data sets; 73.5%). In 36 data sets (22.6%), the reasons for testing were not reported by the authors.

The most common specimen used for iAg testing was nasopharyngeal (‘NP’; 107 data sets, 67.3%). Other studies used combined anterior nasal/mid-turbinate (AN/MT) specimens (35 data sets, 22.0%), saliva (3 data sets, 1.9%), or oropharyngeal (‘OP’; 1 data set, 0.6%) specimens. The specimen type used was unclear in seven studies (13 data sets, 8.1%). Two of the studies pooled nasopharyngeal samples from multiple patients for testing ([51, 52]; also see Supplementary File S2).

Of the 24 unique iAg tests evaluated across all studies, 15 were suitable for POC use and nine were lab-based immunoassays. The most frequently used iAg test was the Sofia SARS Antigen FIA test by Quidel (US; henceforth called Sofia) with 22 data sets (13.8%) and 20,970 (21.8%) tests. The STANDARD F COVID-19 Ag FIA (SD Biosensor Inc., South Korea; henceforth called STANDARD F) was assessed in 18 data sets (11.3%) with 19,617 (20.4%) tests and the BD Veritor System for Rapid Detection of SARS-CoV-2 (Becton, Dickinson and Company [BD], MD, US; henceforth called BD Veritor) in 17 data sets (10.7%) with 11,878 (12.4%) tests, followed by the LumiraDx SARS-CoV-2 Ag test (LumiraDx UK Ltd., UK; henceforth called LumiraDx) with 24 data sets (15.1%) and 10,136 (10.5%) tests. Additional details on each of the iAg tests included in the review are provided in the supplements (Table S1 and File S1).

### Methodological quality of included studies

**Fig. 2:**
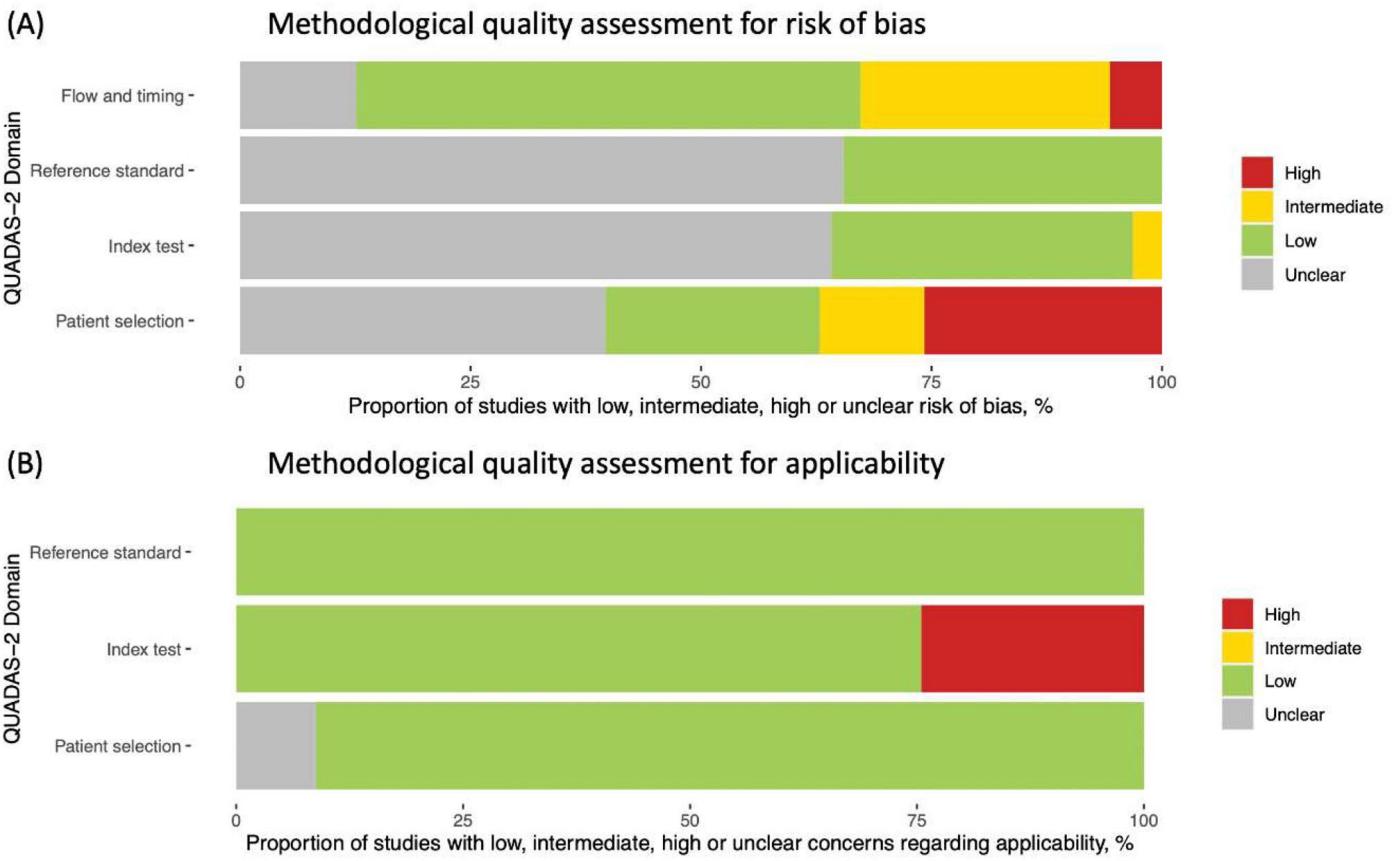
(**A**) QUADAS assessment for risk of bias and (**B**) applicability

The included studies were found to have a variable risk of bias, but high applicability (Fig. 2). Of the data sets evaluated, only 37 (23.3%) data sets included a representative study population by avoiding inappropriate exclusions or a case-control design, resulting in a low risk of bias. A majority of studies were carried out in a routine practice setting, resulting in a high applicability of the included study population to the review in terms of patient selection in a majority of data sets (*n* = 145; 91.2%), while the applicability of the study population was unclear in the remaining data sets (*n* = 14; 8.8%).

The interpretation of the index test results was of low concern for 59 (37.1%) data sets because it was carried out without knowledge of the results of the reference standard; however, the majority of the data sets (*n* = 96; 60.4%) failed to report on the blinded interpretation of the index test results. A predefined threshold was used (*n* = 138; 86.8%) or tests were conducted in accordance with IFU in a majority of the studies (*n* = 120; 75.5%). Index test applicability was judged to be of low concern in 120 (75.5%) data sets, which explicitly mentioned IFU compliance, but high in the remaining 39 (24.5%).

In 104 data sets (65.4%), the reference standard selection, its conduct, or its interpretation was insufficiently described and thus resulted in an unclear risk of bias, which was primarily caused by inadequate reporting of the results' blinded interpretation. The risk of bias in this aspect was low for the remaining data sets (*n* = 55; 34.6%) since the reference standard was administered prior to the iAg tests, and/or the operator administering the reference standard was blinded to the iAg test results, thereby minimizing the potential for bias. The applicability of the reference test was determined to be of low concern for all data sets, because the target condition for this review was defined by viral culture or RT-PCR.

Samples taken simultaneously were used for index and reference testing in 140 (88.1%) of the data sets. In 100 (62.9%) data sets, a single assay was consistently used as the reference, whereas multiple RT-PCR assays were used as the reference in 43 (27.0%) of the data sets (specified in S1). As a result, while also accounting for the possibility that not all patients were included in the analysis, the risk of bias related to flow and timing was assessed to be low in 54.7% of the data sets, intermediate in 27.0%, high in 5.7% and unclear in 12.6%.

The test manufacturers provided financial support for 41 (35.0%) of the studies. In addition, they coauthored 15 of these and two additional studies, accounting for 14.5% of all studies. Moreover, a conflict of interest due to receiving funding from or employment with the test manufacturer was disclosed in 34 studies (29.1%) (File S3).

Analysis of small study effects, which may indicate publication bias, yielded no significant evidence for such effects (*p* = 0.39) (Figure S1).

### Performance of iAg tests in comparison to RT-PCR and/or viral culture

**Fig. 3:**
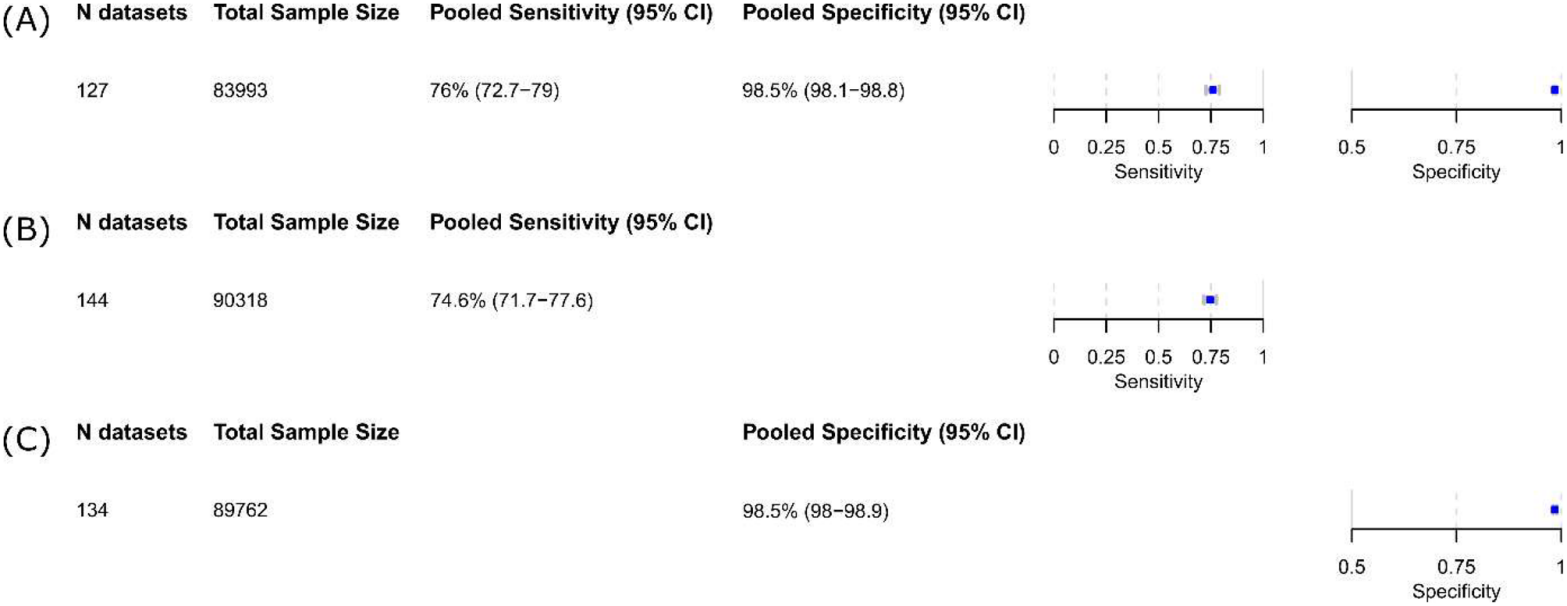
Pooled accuracy of (**A**) bivariant analysis and (**B**) + (**C**) univariant analysis. *Abbreviations* CI = confidence interval

The pooled estimates of sensitivity and specificity for all iAg tests were 76.0% (95% CI 72.7 to 79.0) and 98.5% (95% CI 98.1 to 98.8), respectively, based on the bivariate analysis of the 127 data sets from a total of 99 studies that evaluated 83,993 tests (Fig. 3A, Figure S2). This was slightly higher than a pooled sensitivity of 74.6% (95% CI 71.7 to 77.6) obtained from the univariate analysis of 144 data sets (Fig. 3B, Figure S3). The point estimate of pooled specificity was the same in a univariate analysis of 134 data sets (98.5%; 95% CI 98.0 to 98.9) (Fig. 3C, Figure S4).

**Fig. 4:**
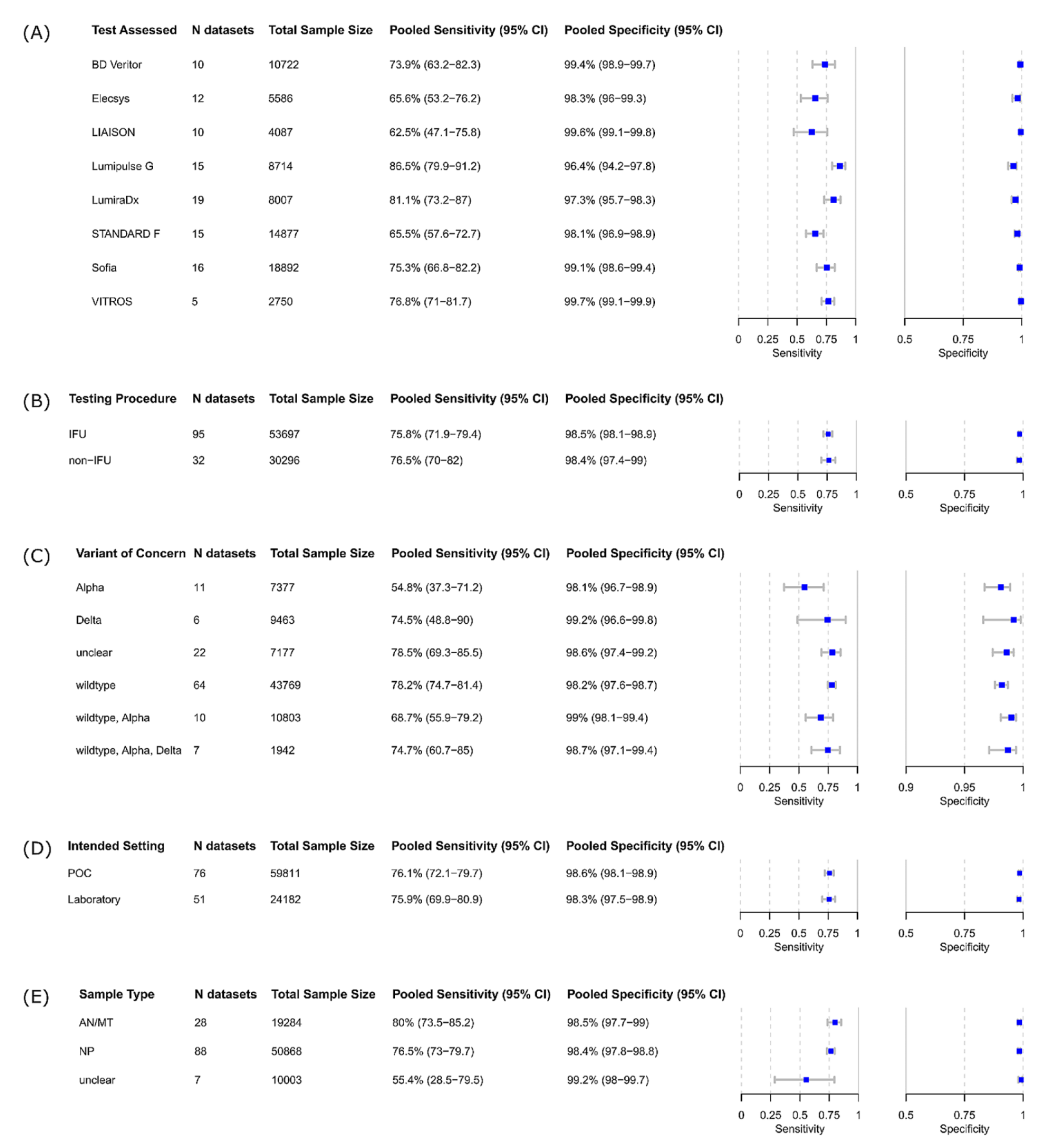
Pooled accuracy of (**A**) per test assessed, (**B**) per IFU-conformity, (**C**) per predominant variant of concern, (**D**) per intended setting, and (**E**) per sample type. *Abbreviations* CI = confidence interval; IFU = instructions for use

Lumipulse G had the highest pooled sensitivity (86.5% [95% CI 79.9 to 91.2]) but the lowest pooled specificity (96.4% [95% CI 94.2 to 97.8]) among the eight tests that were eligible for test-specific meta-analysis (Fig. 4A). LIAISON had the lowest pooled sensitivity (62.5% [95% CI 47.1 to 75.8]). VITROS had the highest pooled specificity at 99.7% (95% CI 99.1 to 99.9). The POC-applicable digital immunoassay BD Veritor had a pooled sensitivity of 73.9% (95% CI 63.2 to 82.3) and a pooled specificity of 99.4% (95% CI 98.9 to 99.7). Among the fluorescence immunoassays (FIAs) with sufficient numbers of data sets (> 4), LumiraDx had the highest pooled sensitivity at 81.1% (95% CI 73.2 to 87.0) but the lowest specificity at 97.3% (95% CI 95.7 to 98.3).

The pooled sensitivity and specificity for IFU-conforming data sets (*n* = 95) were estimated to be 75.8% (95% CI 71.9 to 79.4) and 98.5% (95% CI 98.1 to 98.9), respectively (Fig. 4B). The pooled performance for data sets without reported IFU conformity showed slightly higher sensitivity (76.5%; 95% CI 70.0 to 82.0) and similar specificity (98.4%; 95% CI 97.4 to 99.0).

The highest pooled sensitivity, 78.2% (95% CI 74.7 to 85.5), was observed when the wild-type SARS CoV-2 was predominant (64 data sets, 50.4%) (Fig. 4C). The pooled sensitivity across all studies conducted during a wave of the SARS CoV-2 Alpha variant (11 data sets, 8.7%) was 54.8% (95% CI 37.3 to 71.2), which was the lowest. Based on only six data sets, the pooled sensitivity during the Delta variant was determined to be 74.5% (95% CI 48.8 to 90) with the highest specificity (99.2%; 95% CI 96.6 to 99.8). Only two studies were conducted during the wave of the Omicron variant (2 data sets, 1.6%), with sensitivities ranging from 76.5 to 88.5% [62, 66].

After analyzing the pooled accuracy per intended use setting, the tests intended for lab-based use achieved a sensitivity of 75.9% (95% CI 69.9 to 80.9) and therefore performed similarly to the POC tests, with 76.1% (95% CI 72.1 to 79.7) sensitivity; specificity was almost identical (Fig. 4D).

When only NP samples (88 data sets) were considered, the pooled sensitivity and specificity were estimated to be 76.5% (95% CI 73.0 to 79.7) and 98.4% (95% CI 97.8 to 98.8), respectively (Fig. 4E). Analysis of AN and/or MT samples resulted in a pooled sensitivity of 80.0% with a wide confidence interval (95% CI 73.5 to 85.2) and a pooled specificity of 98.5% (95% CI 97.7 to 99.0).

### Subgroup analyses

#### By age

**Fig. 5:**
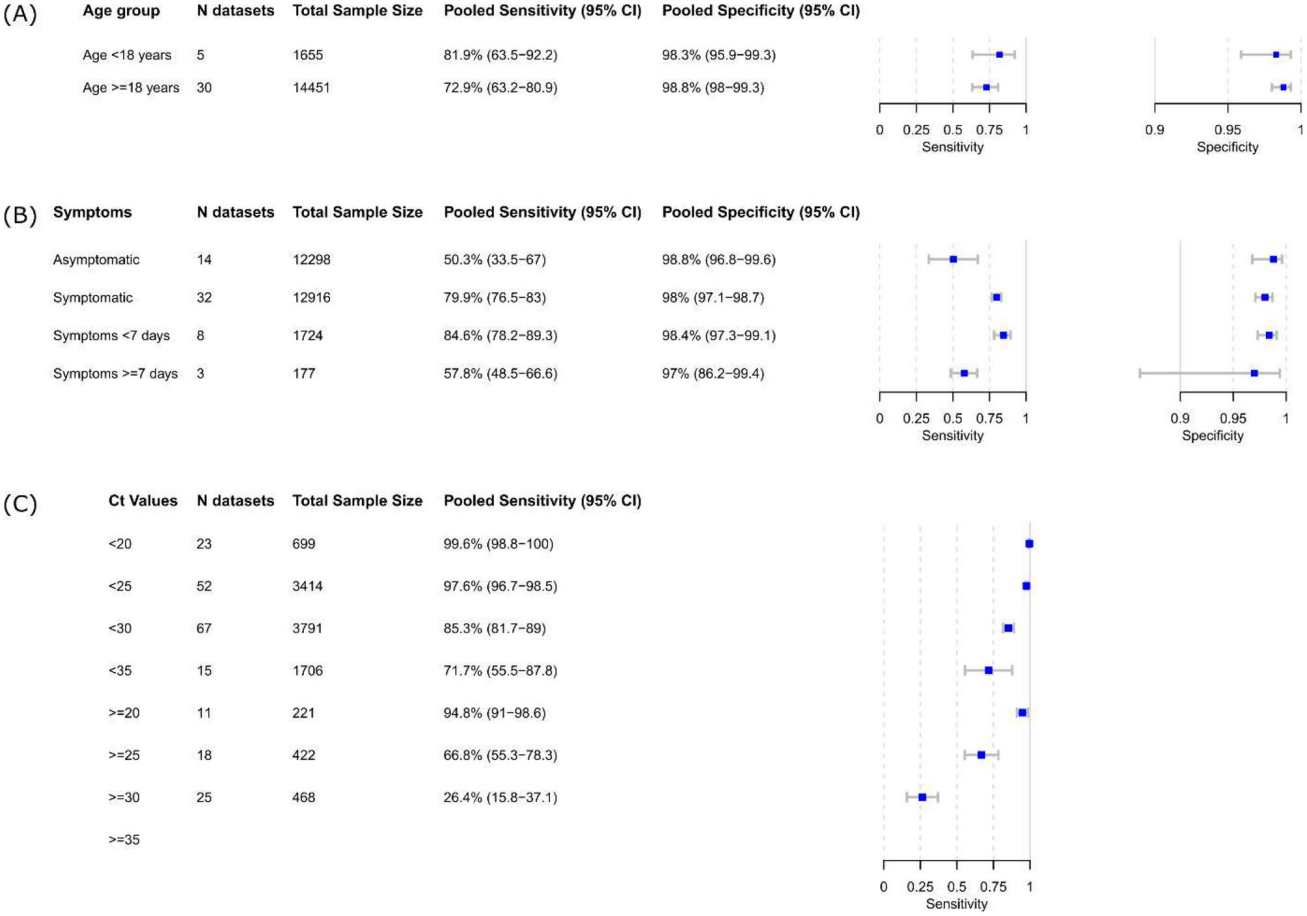
Pooled accuracy of the subgroups (**A**) aged < 18 years and ≥ 18 years, (**B**) symptomatic and asymptomatic persons and (**C**) Ct-values. *Abbreviations* CI = confidence interval; Ct = cycle threshold

Thirty data sets with 14,451 samples from adults (age ≥ 18 years) were available for a meta-analysis, and the results showed a pooled sensitivity and specificity of 72.9% (95% CI 63.2 to 80.9) and 98.8% (95% CI 98.0 to 99.3), respectively (Fig. 5A). Only five datasets with 1,655 samples were available for the pediatric group (age < 18 years) with, compared to adults, a higher pooled sensitivity (81.9%, 95% CI 63.5 to 92.2) and a comparable pooled specificity (98.3%, 95% CI 95.9 to 99.3).

#### By presence of symptoms

Compared to that in the symptomatic group (sensitivity 79.9%; 95% CI 76.5 to 83.0), the pooled sensitivity in the asymptomatic group was substantially lower at 50.3% (95% CI 33.5 to 67.0) (Fig. 5B). Both subgroups had comparably high specificity. As the analysis was repeated per intended use setting, POC tests showed higher sensitivity than the tests intended for lab use in the symptomatic group (81.1% [95% CI 77.6 to 84.1] vs. 69.1% [95% CI 60.8 to 76.4]) (Figure S5A). There were not enough data sets available for the lab tests in the asymptomatic group for the analysis to be repeated (Figure S5B).

#### By duration of symptoms

Data from 1,724 people who were tested within 7 days of the onset of their symptoms were available for the analysis, compared to a very small number of patients (177) who were tested ≥ 7 days after the onset of symptoms (Fig. 5B). In comparison to 84.6% (95% CI 78.2–89.3%) sensitivity for people tested within 7 days of the onset of symptoms, the pooled sensitivity for people tested ≥ 7 days was much lower with only 57.8% (95% CI 48.5–66.6%). The pooled specificity estimates were 98.4% (95% CI 97.3 to 99.1) in the < 7 days group and 97.0% (95% CI 86.2 to 99.4) in the ≥ 7 days group.

#### By Ct values

Fifty-five studies (255 data sets) reported on the performance values based on various Ct value groups, allowing for univariate meta-analysis, which showed that higher Ct values were associated with decreased pooled sensitivity (Fig. 5C). For the Ct value groups < 20 and ≥ 20, the pooled sensitivities were 99.6% (95% CI 98.8 to 100.0) and 94.8% (95% CI 91.0 to 98.6), respectively. For the Ct value group < 25, the pooled sensitivity was 97.8% (95% CI 96.7 to 98.5) but decreased to 85.3% (95% CI 81.7 to 89.0) for the CT value group < 30. The pooled sensitivity for the Ct value group ≥ 30 was estimated to be very low at 26.4% (95% CI 15.8 to 37.1).

### Sensitivity analyses

When case-control studies were excluded the sensitivity and specificity remained similar to the overall pooled sensitivity and specificity estimates with 76.6% (95% CI 72.4 to 80.3) and 98.5% (95% CI 98.0 to 98.9), respectively (Figure S6). Exclusion of preprints did not change the sensitivity or specificity significantly (75.8% [95% CI 72.3 to 79.0] and 98.4% [95% CI 98.0 to 98.8]) (Figure S7). Data from manufacturer-independent studies (68 data sets) produced results with a similar specificity of 98.4% (95% CI 97.8 to 98.8) and a slightly lower sensitivity of 74.4% (95% CI 69.5 to 78.7) (Figure S8).

The studies were also categorized by country income level of the country where participants were enrolled. No significant differences were found between HICs and LMICs for pooled sensitivity (HICs: 75.1%; 95% CI 71.5 to 78.4; LMICs: 76.6%; 95% CI 73.4 to 79.5) or specificity (HICs: 98.6; 95% CI 98.2–98.9; LMICs: 97.1; 95% CI 93.7 to 98.7), with overlapping confidence intervals (Figure S9).

## Discussion

Our systematic review summarized the data from 117 studies that evaluated the clinical accuracy of 24 commercial COVID-19 iAg tests and included a total of 95,181 individuals. The meta-analysis estimated a pooled sensitivity and specificity of 76.0% (95% CI 72.7 to 79.0) and 98.5% (95% CI 98.1 to 98.8), respectively. In a mixed population of symptomatic and asymptomatic individuals, the sensitivity estimate falls short of the WHO’s minimal acceptable sensitivity requirement (≥ 80%), while the pooled specificity exceeded the acceptable specificity requirement (≥ 97%) [4]. One test (LumiraDx) met the requirements for both sensitivity and specificity at 81.1% (95% CI 73.2 to 87.0%) and 97.3% (95% CI 95.7 to 98.3), respectively, aligning with earlier reports [11, 17, 18].

However, when assessing symptomatic individuals within the first week of symptom onset, the pooled performance estimates for all iAg tests satisfied the WHO requirements, indicating the high utility of these tests in this particular population. The lower performance estimates in asymptomatic populations are consistent with previous reports that also considered instrument-free antigen tests [10, 11]. Similarly, subgroup analysis based on Ct values yielded results that were in line with previous studies, indicating that the primary factor influencing test sensitivity is viral load [9, 11, 13, 67]. While the sensitivity estimate for the pediatric group was found to be higher than that of the adult group, we are unable to draw any firm conclusions about how age affects test performance due to the small number of data sets available for the prior age group.

When the wild type was predominant (79.3% [95% CI 75.6 to 82.5]), our subgroup analysis on VoC revealed greater sensitivity than when the Alpha variant was predominant (54.8% [95% CI 37.3 to 71.2]). Notably, out of 11 studies where Alpha predominated, six had unknown symptom statuses, and four had unclear testing timing. Of the 64 studies carried out during the period when the wild type was primarily circulating, 31 reported that testing was conducted regardless of symptoms, and nine did not report on the symptom status. Overall, the substantial interstudy heterogeneity makes it difficult to draw conclusions about performance differences between VoCs. Moreover, due to small sample sizes, the CIs largely overlapped in all variants except the wild type, although this does not rule out a difference between groups.

Overall, we found that the clinical accuracy of the POC and lab-based iAg tests included in the review was comparable. Of note, we estimated the pooled sensitivity of POC-applicable iAg tests to be 76.1% (95% CI 72.1 to 79.7), higher than the 67.1% sensitivity that was previously reported by Keskin et al. [17]. Although there were notably more studies in our review, the overall sample size was smaller. It is also important to note that the analysis of a wide range of lab-based tests with varying analytical sensitivity and potential differences in populations studied may have obscured variations in clinical performance between these platforms.

While our findings indicate that iAg tests alone have no discernible advantages in terms of accuracy over their instrument-free counterparts overall and across all groups as reported in prior systematic reviews [9, 11, 15, 68], iAg tests are still likely to offer benefits from an operational standpoint. Available evidence suggests that errors in the reading and interpretation of instrument-free rapid antigen tests are common and primarily stem from a lack of training and a failure to follow test instructions [69]. Instrument-based POC antigen tests can help decrease human error and subjectivity and, thereby, improve the interpretability of test results. Through their connectivity features, they can also enable automated reporting, which facilitates real-time surveillance; some iAg tests can also potentially increase accessibility, for example for those who are visually impaired. In addition, some lab-based iAg tests have the distinct advantage of offering quick, high-throughput results, making them appropriate for large-scale testing in hospitals and reference laboratories. However, there are infrastructural factors that need to be considered before implementation, such as the availability of trained personnel and laboratory facilities.

To our knowledge, this is the first systematic review that provides a comprehensive summary of the clinical performance of iAg tests for COVID-19 in their intended use settings (laboratory versus POC). The main strengths of our study are the broad search terms we used and the rigorous methodology applied. Additionally, we used an interpretation guide developed *a priori* to assess the methodological quality of the included studies. Moreover, we reported on all literature that was accessible throughout the search period, including preprints and peer-reviewed publications, regardless of language restrictions. There is currently only one other systematic review and meta-analysis that focuses on COVID-19 iAg tests; however, this review is not as comprehensive, does not include a quality assessment, and does not include subgroup analysis [17].

The limitations of this study include the following: (i) Not all commercially available iAg tests were included in our evidence synthesis. As of November 6th, 2023, the FIND COVID-19 test directory lists a total of 85 iAg tests, while we captured only 24 in our study, suggesting that the majority of commercial COVID-19 iAg tests have not been evaluated in published studies [7]. (ii) For the most part, studies that are part of the main analysis either include a mixed population of individuals who are symptomatic and asymptomatic, or they fail to report on the population specifics. As expected, our study confirmed that when symptom status data were available, the sensitivity of iAg tests in symptomatic individuals was substantially higher than that in asymptomatic individuals. Therefore, we cannot completely rule out the possibility that variations in the study populations can account for some of the findings. Similarly, it is unclear whether study participants have received any vaccinations, which may have an effect on their antigen levels and the outcomes of the tests. Additionally, nine studies allowed the use of banked samples, which may have reduced sensitivity estimates in those studies. (iii) In one review study, the reference standard was solely culture [66]. The performance of the index antigen test in this study may have been overestimated due to the lower sensitivity of culture compared to PCR. (iv) The assessment of whether studies followed the manufacturer’s instructions proved to be challenging due to reasons such as poor reporting and version updates. As a result, we relied on whether or not IFU compliance was explicitly stated (in 75% of the studies), which may not be as precise as a comprehensive assessment. (v) As the quality assessment indicated, the shortcomings of the included studies and the fact that there were fewer eligible studies for most individual products limited the scope of our systematic review. By conducting a sensitivity analysis, we aimed to address any data quality constraints caused by the inclusion of preprints in the study. (vi) As previously indicated, the meta-analysis excluded several tests due to the relatively small overall sample size, which limits the significance of the overall results.

## Conclusion

Our systematic review and meta-analysis indicate that commercially available instrument-based antigen diagnostic tests can accurately detect SARS-CoV-2 infections in both laboratory and point-of-care settings, with similar performance estimates to instrument-free antigen tests. As a result, they can have high utility for diagnosing COVID-19 in the early stages of the disease, enabling standardized result interpretation, automated reporting, upscaling test runs and additional advantages such as the simultaneous identification of different pathogens. Choosing which tests to use in clinical settings necessitates a careful evaluation of each product’s performance, as confirmed by independent studies and operational features.

### Electronic supplementary material

Below is the link to the electronic supplementary material.

Supplementary Material 1

Supplementary Material 2

Supplementary Material 3

Supplementary Material 4

## Data Availability

Data is provided within the manuscript or supplementary information files.

## Abbreviations

Ag-RDT: Antigen detection rapid diagnostic test
AN: Anterior nasal
CI: Confidence interval
CLEIA: Chemiluminescence enzyme immunoassay
CLIA: Chemiluminescence immunoassay
Ct: Cycle threshold
DIA: Diffusion immunoassay
DOS: Duration of symptoms
ECLIA: Electrochemiluminescence immunoassay
ELISA: Enzyme-linked immunosorbent assay
FIA: Fluorescence immunoassay
FN: False negative
FP: False positive
HIC: High-income countries
iAg test: Instrument-based antigen test
IFU: Instructions for use
MIC: Middle-income countries
NAATs: Nucleic acid amplification tests
NMT: Nasal mid-turbinate
NP: Nasopharyngeal
NPA: Negative percentage agreement
OP: Oropharyngeal
POC: Point of care
PRISMA: Preferred Items for Systematic Reviews and Meta-analysis
RCT: Randomized controlled trial
RT-PCR: Reverse transcription polymerase chain reaction
TN: True negative
TP: True positive
VoC: Variant of Concern
WHO: World Health Organization
