# Supplementary material for: Clinical accuracy of instrument-based SARS-CoV-2 antigen diagnostic tests: a systematic review and meta-analysis
Source: Virol J. 2024 Apr 29;21:99. doi: 10.1186/s12985-024-02371-5 (PMC11059670; doi:10.1186/s12985-024-02371-5)
Supplement: Supplementary file 1 — Supplementary Material 1 [file 12985_2024_2371_MOESM1_ESM.docx]

**S3 – Studies potentially influenced by the test manufacturer (number of studies: 51)**

1. a4, [Eric T Beck](https://pubmed.ncbi.nlm.nih.gov/?term=Beck+ET&cauthor_id=33239376), et al. Comparison of Quidel Sofia SARS FIA Test to Hologic Aptima SARS-CoV-2 TMA Test for Diagnosis of COVID-19 in Symptomatic Outpatients. J Clin Microbiol. 2021 Jan 21;59(2):e02727-20. DOI: [10.1128/JCM.02727-20](https://doi.org/10.1128/jcm.02727-20)

2. a 28, Pekosz A, Cooper C, Parvu V, Li M, Andrews J, Manabe YCC, et al. Antigen-based testing but not real-time PCR correlates with SARS-CoV-2 virus culture. Clinical Infectious Diseases, 2020; ciaa1706. DOI:10.1093/cid/ciaa1706.

3. a32, Lorena Porte, et al. Evaluation of two fluorescence immunoassays for the rapid detection of SARS-CoV-2 antigen - new tool to detect infective COVID-19 patients; [PeerJ.](https://www.ncbi.nlm.nih.gov/pmc/articles/PMC7827970/) 2021; 9: e10801. 2021 Jan 21. doi: [10.7717/peerj.10801](https://doi.org/10.7717%2Fpeerj.10801)

4. a41, Thomas Weitzel, et al. Comparative evaluation of four rapid SARS-CoV-2 antigen detection tests using universal transport medium; Travel Med Infect Dis. 2021 Jan-Feb;39:101942. DOI: 10.1016/j.tmaid.2020.101942.

5. a43, Young S, Taylor SN, Cammarata CL, Varnado KG, Roger-Dalbert C, Montano A, et al. Clinical evaluation of BD Veritor SARS-CoV-2 point-of-care test performance compared to PCR-based testing and versus the Sofia 2 SARS Antigen point-of-care test. Journal of Clinical Microbiology, 2020; 59(1). DOI:10.1128/jcm.02338-20.

6. f20, Osterman A, Baldauf HM, Eletreby M, Wettengel JM, Afridi SQ, Fuchs T, et al. Evaluation of two rapid antigen tests to detect SARS-CoV-2 in a hospital setting. Medical Microbiology and Immunology, 2021; 210(1):65-72. DOI:10.1007/s00430-020-00698-8.

7. f43, Drain PK, Ampajwala M, Chappel C, Gvozden AB, Hoppers M, Wang M, et al. A Rapid, High-Sensitivity SARS-CoV-2 Nucleocapsid Immunoassay to Aid Diagnosis of Acute COVID-19 at the Point of Care: A Clinical Performance Study. Infectious Diseases and Therapy, 2021; 10(2):753–761. DOI:10.1007/s40121-021-00413-x.

8. f70, Stefanie Lefever, et al. Comparison of the quantitative DiaSorin Liaison antigen test to RT-PCR for the diagnosis of COVID-19 in symptomatic and asymptomatic outpatients. J Clin Microbiol. 2021 Jun 18;59(7):e0037421. doi: 10.1128/JCM.00374-21.

9. f74, Nobuhiro Asai, et al. Efficacy and validity of automated quantitative chemiluminescentenzyme immunoassay for SARS-CoV-2 antigen test from salivaspecimen in the diagnosis of COVID-19. J Infect Chemother. 2021 Jul;27(7):1039-1042. doi: 10.1016/j.jiac.2021.03.021.

10. f77, Micocci M, Buckle P, Hayward G, Allen J, Davies K, Kierkegaard P, et al. Point of Care Testing using rapid automated Antigen Testing for SARS-COV-2 in Care Homes – an exploratory safety, usability and diagnostic agreement evaluation. medRxiv [Preprint]; published April 26, 2021. DOI:10.1101/2021.04.22.21255948.

11. f78, Dominik Nörz, et al. Multicenter evaluation of a fully automated high-throughput SARS-CoV-2 antigen immunoassay; Infect Dis Ther. 2021 Dec;10(4):2371-2379. DOI: 10.1007/s40121-021-00510-x.

12. f82, Yin N, Debuysschere C, Decroly M, Bouazza FZ, Collot V, Martin C, et al. SARS-CoV-2 Diagnostic Tests: Algorithm and Field Evaluation From the Near Patient Testing to the Automated Diagnostic Platform. Frontiers in Medicine, 2021; 8:380. DOI:10.3389/fmed.2021.650581.

13. f109, Christensen K, Ren H, Chen S, Cooper C, Young S. Clinical evaluation of BD Veritor™ SARS-CoV-2 and Flu A+B Assay for point-of-care (POC) System. medRxiv [Preprint]; published May 05, 2021. DOI:10.1101/2021.05.04.21256323.

14. f113, Blairon L, Cupaiolo R, Thomas I, Piteüs S, Wilmet A, Beukinga I, et al. Efficacy comparison of three rapid antigen tests for SARS-CoV-2 and how viral load impact their performance. Journal of Medical Virology, 2021; 93:5783-5788. DOI:10.1002/jmv.27108.

15. f128, Mercedes Domínguez Fernández, et al. Usefulness of the Lumiradx™ SARS-COV-2 antigen test in nursing home; Enferm Infecc Microbiol Clin (Engl Ed). 2022 Aug-Sep;40(7):404-405. DOI: 10.1016/j.eimce.2022.04.008.

16. f148, Karon BS, Donato L, Bridgeman AR, Blommel JH, Kipp B, Maus A, et al. Analytical sensitivity and specificity of four point of care rapid antigen diagnostic tests for SARS-CoV-2 using real-time quantitative PCR, quantitative droplet digital PCR, and a mass spectrometric antigen assay as comparator methods. Clinical Chemistry, 2021; hvab138. DOI:10.1093/clinchem/hvab138.

17. f154, Bachman CM, Grant BD, Anderson CE, Alonzo LF, Garing S, Byrnes SA, et al. Clinical validation of an open-access SARS-COV-2 antigen detection lateral flow assay, compared to commercially available assays. PLoS ONE, 2021; 16(8):e0256352. DOI:10.1371/journal.pone.0256352.

18. f165, Baccani I, Morecchiato F, Chilleri C, Cervini C, Gori E, Matarrese D, et al. Evaluation of Three Immunoassays for the Rapid Detection of SARS-CoV-2 Antigens. Diagnostic Microbiology and Infectious Disease, 2021; 101(2):115434. DOI:10.1016/j.diagmicrobio.2021.115434.

19. f171, Bilal Iqbal, et al. Comparison of SARS-CoV-2 antigen electrochemiluminescence immunoassay to RT-PCR assay for laboratory diagnosis of COVID-19 in Peshawar; Diagnosis (Berl). 2021 Aug 30;9(3):364-368. doi: 10.1515/dx-2021-0078.

20. f184, Anette Audigé, et al. Reduced Relative Sensitivity of the Elecsys SARS-CoV-2 Antigen Assay in Saliva Compared to Nasopharyngeal Swabs; Microorganisms. 2021 Aug 10;9(8):1700. doi: 10.3390/microorganisms9081700.

21. f188, Nikolay Mayanskiy, et al. Parallel detection of SARS-CoV-2 RNA and nucleocapsid antigen in nasopharyngeal specimens from a COVID-19 patient screening cohort; Int J Infect Dis. 2021 Jul;108:330-332. doi: 10.1016/j.ijid.2021.05.082.

22. f191, Orsi A, Pennati BM, Bruzzone B, Ricucci V, Ferone D, Barbera P, et al. On-field evaluation of a ultra-rapid fluorescence immunoassay as a frontline test for SARS-COV-2 diagnostic. Journal of Virological Methods, 2021; 295:114201. DOI:10.1016/j.jviromet.2021.114201.

23. f192, Lori Bourassa, et al. A SARS-CoV-2 Nucleocapsid Variant that Affects Antigen Test Performance; J Clin Virol. 2021 Aug;141:104900. doi: 10.1016/j.jcv.2021.104900.

24. f194, Suzuki H, Akashi Y, Ueda A, Kiyasu Y, Takeuchi Y, Maehara Y, et al. Diagnostic performance of a novel digital immunoassay (RapidTesta SARS-CoV-2): a prospective observational study with 1,127 nasopharyngeal samples. medRxiv [Preprint]; published August 04, 2021. DOI:10.1101/2021.07.26.21261162.

25. f197, Juha M Koskinen, et al. Clinical validation of automated and rapid mariPOC SARS-CoV-2 antigen test; Sci Rep. 2021 Oct 13;11(1):20363.

 DOI: 10.1038/s41598-021-99886-6.

26. k1, Hannah Wang, et al. Ultra-sensitive Severe Acute Respiratory Syndrome Coronavirus 2 (SARS-CoV-2) Antigen Detection for the Diagnosis of Coronavirus Disease 2019 (COVID-19) in Upper Respiratory Samples; Clin Infect Dis. 2021 Dec 16;73(12):2326-2328. doi: 10.1093/cid/ciab063.

27. k4, Myriam Ben Abdelhanin, et al. Evaluation of the Elecsys SARS-CoV-2 antigen assay for the detection of SARS-CoV-2 in nasopharyngeal swabs; J Clin Virol. 2021 Nov; 144: 104991.2021 Oct 1. doi: 10.1016/j.jcv.2021.104991

28. k8, MD Paul Drain, et al. Performance of the LumiraDx Microfluidic Immunofluorescence Point-of-Care SARS-CoV-2 Antigen Test in Asymptomatic Adults and Children; Am J Clin Pathol. 2022 Apr; 157(4): 602–607. 2021 Oct 20. doi: 10.1093/ajcp/aqab173

29. k12, Stephanie L Mitchell, et al. Performance of SARS-CoV-2 antigen testing in symptomatic and asymptomatic adults: a single-center evaluation; BMC Infect Dis. 2021 Oct 18;21(1):1071. doi: 10.1186/s12879-021-06716-1.

30. k15, Giuseppe Sberna, et al. Comparison of Allplex™ SARS-CoV-2 Assay, Easy SARS-CoV-2 WE and Lumipulse quantitative SARS-CoV-2 antigen test performance using automated systems for the diagnosis of COVID-19; Int J Infect Dis. 2021 Dec;113:113-115. doi: 10.1016/j.ijid.2021.09.069.

31. k19, Nira R Pollock, et al. Correlation of SARS-CoV-2 Nucleocapsid Antigen and RNA Concentrations in Nasopharyngeal Samples from Children and Adults Using an Ultrasensitive and Quantitative Antigen Assay; J Clin Microbiol. 2021 Mar 19;59(4):e03077-20. doi: 10.1128/JCM.03077-20.

32. s1, Amanda Agard, et al. Clinical comparison and agreement of PCR, antigen, and viral culture for the diagnosis of COVID-19: Clinical Agreement Between Diagnostics for COVID19; J Clin Virol Plus. 2022 Aug;2(3):100099. doi: 10.1016/j.jcvp.2022.100099.

33. s3, Mohammad Alghounaim, et al. The Performance of Two Rapid Antigen Tests During Population-Level Screening for SARS-CoV-2 Infection; Front Med (Lausanne). 2021 Dec 23;8:797109. doi: 10.3389/fmed.2021.797109.

34. s10, Slim Fourati, et al. Performance of a high-throughput, automated enzyme immunoassay for the detection of SARS-CoV-2 antigen, including in viral "variants of concern": Implications for clinical use; J Clin Virol. 2022 Jan;146:105048. doi: 10.1016/j.jcv.2021.105048.

35. s11, Megan Culler Freeman, et al. Performance of the Sofia SARS-CoV-2 Rapid Antigen Test in Symptomatic and Asymptomatic Pediatric Patients; J Pediatric Infect Dis Soc. 2022 Sep 29;11(9):417-421. doi: 10.1093/jpids/piac035.

36. s15, Aurélie Gourgeon, et al. Performance of 22 Rapid Lateral Flow Tests for SARS-CoV-2 Antigen Detection and Influence of "Variants of Concern": Implications for Clinical Use; Microbiol Spectr. 2022 Aug 31;10(4):e0115722. doi: 10.1128/spectrum.01157-22.

37. s16, Gilbert Greub, et al. Multicenter Technical Validation of 30 Rapid Antigen Tests for the Detection of SARS-CoV-2 (VALIDATE); Microorganisms. 2021 Dec; 9(12): 2589. 2021 Dec 15. doi: 10.3390/microorganisms9122589

38. s21, James E Kirby, et al. Sars-Cov-2 antigen tests predict infectivity based on viral culture: comparison of antigen, PCR viral load, and viral culture testing on a large sample cohort; Clin Microbiol Infect. 2023 Jan;29(1):94-100. doi: 10.1016/j.cmi.2022.07.010.

39. s23, Gema Fernández-Rivas, et al. Analytical Performance of Quantitative DiaSorin Liaison SARS-COV-2 Antigen Test for the Asymptomatic Population; Front Public Health. 2022 Jan 7;9:788581. doi: 10.3389/fpubh.2021.788581.

40. s26, Chin Shern Lau, et al. Evaluation and Validation of the Roche Elecsys SARS-CoV-2 Antigen Electro-Chemiluminescent Immunoassay in a Southeast Asian Region; Vaccines (Basel). 2022 Jan 27;10(2):198. doi: 10.3390/vaccines10020198.

41. s27, Joachim Linssen, et al. A method comparison study of the high throughput automated HISCL® SARS-CoV-2 antigen assay using nasopharyngeal swab samples from symptomatic and asymptomatic subjects against conventional RT-PCR; J Med Virol. 2022 Jul;94(7):3070-3080. doi: 10.1002/jmv.27679.

42. s29, Mateusz Miłosz, et al. Validation of the test for detecting SARS--CoV-2 antigens in the Polish population in patients with suspected SARS-CoV-2 infection; Cent Eur J Immunol. 2022; 47(1): 58–62. 2022 Feb 28. doi: 10.5114/ceji.2022.113992

43. s33, Hidetoshi Nomoto, et al. Potential usage of anterior nasal sampling in clinical practice with three rapid antigen tests for SARS-CoV-2; J Infect Chemother. 2023 Jan;29(1):15-19. doi: 10.1016/j.jiac.2022.09.001.

44. s35, Octavia Peck Palmer, et al. Performance of High Throughput SARS-CoV-2 Antigen Testing Compared to Nucleic Acid Testing; Lab Med. 2023 Mar 7;54(2):e54-e57. doi: 10.1093/labmed/lmac107.

45. s36, Dorian Petonnet, et al. Comparison of Rapid and Automated Antigen Detection Tests for the Diagnosis of SARS-CoV-2 Infection; Diagnostics (Basel). 2022 Jan 4;12(1):104. doi: 10.3390/diagnostics12010104.

46. s37, Matthew L. Robinson, et al. Limitations of Molecular and Antigen Test Performance for SARS-CoV-2 in Symptomatic and Asymptomatic COVID-19 Contacts; ASM Journals 2022 June; DOI: <https://doi.org/10.1128/jcm.00187-22>

47. s48, Stephanie Uster, et al. Evaluation of the DiaSorin LIAISON SARS-CoV-2 antigen assay on nasopharyngeal swabs in two different SARS-CoV-2 pandemic waves in Switzerland: The impact of the Omicron variant on its performance; J Clin Virol Plus. 2022 Aug;2(3):100095. doi: 10.1016/j.jcvp.2022.100095.

48. s49, Chad R Wells, et al. Comparative analyses of eighteen rapid antigen tests and RT-PCR for COVID-19 quarantine and surveillance-based isolation; Commun Med (Lond). 2022 Jul 9;2:84. doi: 10.1038/s43856-022-00147-y.

49. s51, Kyunghee Yu, et al. Clinical Evaluation of Two Rapid Antigen Tests for Severe Acute Respiratory Syndrome Coronavirus 2 Detection; Ann Lab Med. 2023 Jan 1;43(1):120-123. doi: 10.3343/alm.2023.43.1.120.

50. s28, Amrish Mehta, et al. Performance of Gazelle COVID-19 point-of-care test for detection of nucleocapsid antigen from SARS-CoV-2; Preprint from medRxiv, 23 Mar 2022 DOI: 10.1101/2022.03.23.22272094

51. s39, Kaori Saito, et al. Performance and usefulness of a novel automated immunoassay HISCL SARS‐CoV‐2 Antigen assay kit for the diagnosis of COVID‐19 Sci Rep. 2021 Dec 1;11(1):23196. doi: 10.1038/s41598-021-02636-x.
