# Supplementary material for: Clinical accuracy of instrument-based SARS-CoV-2 antigen diagnostic tests: a systematic review and meta-analysis
Source: Virol J. 2024 Apr 29;21:99. doi: 10.1186/s12985-024-02371-5 (PMC11059670; doi:10.1186/s12985-024-02371-5)
Supplement: Supplementary file 2 — Supplementary Material 2 [file 12985_2024_2371_MOESM2_ESM.docx]

**Supplementary File S1**

**Clinical accuracy of instrument-based SARS-CoV-2 antigen diagnostic tests: A systematic review and meta-analysis**

Katharina Manten^1,2^, Stephan Katzenschlager^1^, Lukas E. Brümmer^2^, Stephani Schmitz^2,3^, Mary Gaeddert^2^, Christian Erdmann^4^, Maurizio Grilli^5^, Nira R. Pollock^6,^ Aurélien Macé^7^, Berra Erkosar^7^, Sergio Carmona^7^, Stefano Ongarello^7^, Cheryl C. Johnson^8^, Jilian A. Sacks^9^, Verena Faehling^2^, Linus Bornemann^10^, Markus A. Weigand^1^, Claudia M. Denkinger^2, 11^,

Seda Yerlikaya^2,11^*

* Corresponding author

[seda.yerlikaya@uni-heidelberg.de](mailto:seda.yerlikaya@uni-heidelberg.de)

Department of Infectious Disease and Tropical Medicine, Heidelberg University Hospital

Im Neuenheimer Feld 324

69120 Heidelberg, Germany

1) Department of Anesthesiology, Heidelberg University Hospital, Heidelberg, Germany

2) Department of Infectious Disease and Tropical Medicine, Heidelberg University Hospital, Heidelberg, Germany

3) Department of Developmental Biology, Erasmus Medical Center, Rotterdam, Netherlands

4) FH Muenster University of Applied Sciences, Muenster, Germany

5) Library, University Medical Center Mannheim, Mannheim, Germany

6) Department of Laboratory Medicine, Boston Children’s Hospital, Boston, Massachusetts, United States of America

7) FIND, Geneva, Switzerland

8) Global HIV, Hepatitis and STIs Programmes, World Health Organization, Geneva, Switzerland

9) Department of Epidemic and Pandemic Preparedness and Prevention, World Health Organization, Geneva, Switzerland

10) Institute of Virology, University Medical Centre and Faculty of Medicine, University of Freiburg, Freiburg, Germany

11) German Center for Infection Research (DZIF), partner site Heidelberg University Hospital, Heidelberg, Germany

Table of content

[Supplement PRISMA Checklist 4](#_Toc159790865)

[Supplement Text 1 – Search Strategy 9](#_Toc159790866)

[Review questions 9](#_Toc159790867)

[Definitions 9](#_Toc159790868)

[Strategie 9](#_Toc159790869)

[Searched Databases 9](#_Toc159790870)

[PubMed 9](#_Toc159790871)

[Web of Science Core Collection 10](#_Toc159790872)

[Bio_MedRxiv 11](#_Toc159790873)

[Supplement Text 2 – Supplementary Methods 12](#_Toc159790874)

[Supplement Text 3 – Definitions and underlying Technologies 14](#_Toc159790875)

# Supplement PRISMA Checklist

| **Section and Topic** | **Item #** | **Checklist item** | **Location where item is reported** |
| --- | --- | --- | --- |
| **TITLE** | | |  |
| Title | 1 | Identify the report as a systematic review. | Title of paper |
| **ABSTRACT** | | |  |
| Abstract | 2 | See the PRISMA 2020 for Abstracts checklist. | Abstract Checklist |
| **INTRODUCTION** | | |  |
| Rationale | 3 | Describe the rationale for the review in the context of existing knowledge. | Introduction |
| Objectives | 4 | Provide an explicit statement of the objective(s) or question(s) the review addresses. | Introduction |
| **METHODS** | | |  |
| Eligibility criteria | 5 | Specify the inclusion and exclusion criteria for the review and how studies were grouped for the syntheses. | Subsection “Eligibility criteria” |
| Information sources | 6 | Specify all databases, registers, websites, organisations, reference lists and other sources searched or consulted to identify studies. Specify the date when each source was last searched or consulted. | Subsection “Search strategy” |
| Search strategy | 7 | Present the full search strategies for all databases, registers and websites, including any filters and limits used. | Supplementary Material “S1 Text Search Strategy” |
| Selection process | 8 | Specify the methods used to decide whether a study met the inclusion criteria of the review, including how many reviewers screened each record and each report retrieved, whether they worked independently, and if applicable, details of automation tools used in the process. | Selection process was done accordingly to our previous analysis. Subsection “Study Selection, Data Extraction and Assessment of Independence from Manufacturers” |
| Data collection process | 9 | Specify the methods used to collect data from reports, including how many reviewers collected data from each report, whether they worked independently, any processes for obtaining or confirming data from study investigators, and if applicable, details of automation tools used in the process. | Data collection process was done accordingly to our previous analysis. Subsection “Study Selection, Data Extraction and Assessment of Independence from Manufacturers” |
| Data items | 10a | List and define all outcomes for which data were sought. Specify whether all results that were compatible with each outcome domain in each study were sought (e.g. for all measures, time points, analyses), and if not, the methods used to decide which results to collect. | Supplementary File “S2 Table Parameters” |
|  | 10b | List and define all other variables for which data were sought (e.g. participant and intervention characteristics, funding sources). Describe any assumptions made about any missing or unclear information. | Supplementary Files S2 Table and https://doi.org/10.11588/data/P9JEPG |
| Study risk of bias assessment | 11 | Specify the methods used to assess risk of bias in the included studies, including details of the tool(s) used, how many reviewers assessed each study and whether they worked independently, and if applicable, details of automation tools used in the process. | Study risk of bias assessment was done accordingly to our previous analysis. Methods section.  Subsection “Assessment of methodological quality” |
| Effect measures | 12 | Specify for each outcome the effect measure(s) (e.g. risk ratio, mean difference) used in the synthesis or presentation of results. | Methods section. Subsection “Statistical analysis and data synthesis” |
| Synthesis methods | 13a | Describe the processes used to decide which studies were eligible for each synthesis (e.g. tabulating the study intervention characteristics and comparing against the planned groups for each synthesis (item #5)). | Methods section. Subsection “Eligibility Criteria” |
|  | 13b | Describe any methods required to prepare the data for presentation or synthesis, such as handling of missing summary statistics, or data conversions. | Methods section. Subsection “Study Selection and Data Extraction” and “Statistical analysis and data synthesis” |
|  | 13c | Describe any methods used to tabulate or visually display results of individual studies and syntheses. | Methods section. Subsection “Statistical analysis and data synthesis” |
|  | 13d | Describe any methods used to synthesize results and provide a rationale for the choice(s). If meta-analysis was performed, describe the model(s), method(s) to identify the presence and extent of statistical heterogeneity, and software package(s) used. | Methods section. Subsection “Statistical analysis and data synthesis” |
|  | 13e | Describe any methods used to explore possible causes of heterogeneity among study results (e.g. subgroup analysis, meta-regression). | Methods section. Subsection “Statistical analysis and data synthesis” |
|  | 13f | Describe any sensitivity analyses conducted to assess robustness of the synthesized results. | Methods section. Subsection “Statistical analysis and data synthesis” and Results section “Sensitivity analysis” |
| Reporting bias assessment | 14 | Describe any methods used to assess risk of bias due to missing results in a synthesis (arising from reporting biases). | Methods section. Subsection “Statistical analysis and data synthesis” |
| **RESULTS** | | |  |
| Study selection | 16a | Describe the results of the search and selection process, from the number of records identified in the search to the number of studies included in the review, ideally using a flow diagram. | Fig1 PRISMA Flow Diagram |
|  | 16b | Cite studies that might appear to meet the inclusion criteria, but which were excluded, and explain why they were excluded. | Section Results, Paragraph 1 and Figure 1 |
| Study characteristics | 17 | Cite each included study and present its characteristics. | Supplementary Files S2 and https://doi.org/10.11588/data/P9JEPG |
| Risk of bias in studies | 18 | Present assessments of risk of bias for each included study. | Figure 2 QUADAS and https://doi.org/10.11588/data/P9JEPG |
| Results of individual studies | 19 | For all outcomes, present, for each study: (a) summary statistics for each group (where appropriate) and (b) an effect estimate and its precision (e.g. confidence/credible interval), ideally using structured tables or plots. | Subsection “Study description” and Figure 3 and File S2 |
| Results of syntheses | 20a | For each synthesis, briefly summarise the characteristics and risk of bias among contributing studies. | Subsection “Methodological quality of included studies” and Figure 2A |
|  | 20b | Present results of all statistical syntheses conducted. If meta-analysis was done, present for each the summary estimate and its precision (e.g. confidence/credible interval) and measures of statistical heterogeneity. If comparing groups, describe the direction of the effect. | Subsection “Performance of iAg tests in comparison to RT-PCR and/or viral culture” and Figure 3  Subsection “Subgroup analyses” and Figure 4+5, Supplementary File S2 |
|  | 20c | Present results of all investigations of possible causes of heterogeneity among study results. | Subsections “IFU conformity”, “Presence of symptoms”, “Duration of Symptoms”, “Ct Values”, “Age”, “VoC”, and “LMIC vs. HIC” |
|  | 20d | Present results of all sensitivity analyses conducted to assess the robustness of the synthesized results. | Subsection “Sensitivity Analysis” |
| Reporting biases | 21 | Present assessments of risk of bias due to missing results (arising from reporting biases) for each synthesis assessed. | Subsection “Statistical analysis and data synthesis”, “Publication bias” Figure 6 https://doi.org/10.11588/data/P9JEPG |
| Certainty of evidence | 22 | Present assessments of certainty (or confidence) in the body of evidence for each outcome assessed. | Subsection “Statistical analysis and data synthesis” |
| **DISCUSSION** | | |  |
| Discussion | 23a | Provide a general interpretation of the results in the context of other evidence. | Paragraph 1-4 |
|  | 23b | Discuss any limitations of the evidence included in the review. | Paragraph 6 |
|  | 23c | Discuss any limitations of the review processes used. | Paragraph 6 |
|  | 23d | Discuss implications of the results for practice, policy, and future research. | Section “Conclusion” |
| **OTHER INFORMATION** | | |  |
| Registration and protocol | 24a | Provide registration information for the review, including register name and registration number, or state that the review was not registered. | Abstract, Section “Methods” First Paragraph |
|  | 24b | Indicate where the review protocol can be accessed, or state that a protocol was not prepared. | Abstract, Section “Methods” and Supplementary Files “Text Study Protocol” in former reviews |
|  | 24c | Describe and explain any amendments to information provided at registration or in the protocol. | N/A |
| Support | 25 | Describe sources of financial or non-financial support for the review, and the role of the funders or sponsors in the review. | Section Statement and declarations: “Funding” |
| Competing interests | 26 | Declare any competing interests of review authors. | N/A |
| Availability of data, code and other materials | 27 | Report which of the following are publicly available and where they can be found: template data collection forms; data extracted from included studies; data used for all analyses; analytic code; any other materials used in the review. | Data is available under File S1 |

*From:*  Page MJ, McKenzie JE, Bossuyt PM, Boutron I, Hoffmann TC, Mulrow CD, et al. The PRISMA 2020 statement: an updated guideline for reporting systematic reviews. BMJ 2021;372:n71. doi: 10.1136/bmj.n71

For more information, visit: <http://www.prisma-statement.org/>

**Prisma 2020 for Abstracts Checklist**

| **Section and Topic** | **Item #** | **Checklist item** | **Reported (Yes/No)** |
| --- | --- | --- | --- |
| **TITLE** | | |  |
| Title | 1 | Identify the report as a systematic review. | Yes |
| **BACKGROUND** | | |  |
| Objectives | 2 | Provide an explicit statement of the main objective(s) or question(s) the review addresses. | Yes |
| **METHODS** | | |  |
| Eligibility criteria | 3 | Specify the inclusion and exclusion criteria for the review. | Yes |
| Information sources | 4 | Specify the information sources (e.g. databases, registers) used to identify studies and the date when each was last searched. | Yes |
| Risk of bias | 5 | Specify the methods used to assess risk of bias in the included studies. | Yes |
| Synthesis of results | 6 | Specify the methods used to present and synthesise results. | Yes |
| **RESULTS** | | |  |
| Included studies | 7 | Give the total number of included studies and participants and summarise relevant characteristics of studies. | Yes |
| Synthesis of results | 8 | Present results for main outcomes, preferably indicating the number of included studies and participants for each. If meta-analysis was done, report the summary estimate and confidence/credible interval. If comparing groups, indicate the direction of the effect (i.e. which group is favoured). | Yes |
| **DISCUSSION** | | |  |
| Limitations of evidence | 9 | Provide a brief summary of the limitations of the evidence included in the review (e.g. study risk of bias, inconsistency and imprecision). | Yes |
| Interpretation | 10 | Provide a general interpretation of the results and important implications. | Yes |
| **OTHER** | | |  |
| Funding | 11 | Specify the primary source of funding for the review. | No |
| Registration | 12 | Provide the register name and registration number. | Yes |

*From:*  Page MJ, McKenzie JE, Bossuyt PM, Boutron I, Hoffmann TC, Mulrow CD, et al. The PRISMA 2020 statement: an updated guideline for reporting systematic reviews. BMJ 2021;372:n71. doi: 10.1136/bmj.n71 For more information, visit: <http://www.prisma-statement.org/>

# Supplement Text 1 – Search Strategy

## Review questions

To assess the accuracy and ease-of-use of marketable antigen point of care diagnostics for SARS-CoV-2 compared to RT-PCR based on manufacturer independent evaluations.

Restriction: start Dezember 2019

## Definitions

P

| SARS-CoV-2 |
| --- |

I

| Antigen nachweisenden Schnelltests Ag RDT |
| --- |

## Strategie

| 1 | P |
| --- | --- |
| 2 | I |
| 3 | 1 AND 2 |

## Searched Databases

- PubMed
- Web of Science Core Collection
- BioRxiv
- MedRxiv

## PubMed

P

| (**"Severe Acute Respiratory Syndrome Coronavirus 2"[Supplementary Concept] OR**  **"COVID-19" [Supplementary Concept] OR**  **"Betacoronavirus"[Mesh] OR**  **"Coronavirus"[Mesh] OR**  covid*[tw] OR  "coronavirus*"[tw] OR  "corona virus*"[tw] OR  ncov*[tw] OR  "n cov*"[tw] OR  sarscov*[tw] OR  "sars cov*"[tw] OR  "2019nCoV*"[tw] OR  "2019 nCoV*"[tw] OR  "sars2*"[tw] OR  "sars 2*"[tw]) |
| --- |

I

| **"Point-of-Care Testing"[Mesh] OR**  Antigen[tw] OR  “Lateral flow”[tw] OR  RDT[tw] OR  (("Point of Care*"[tw] OR  "Bedside*"[tw] OR  Rapid*[tw])  AND  Test*[tw]) |
| --- |

**P**

**1 (99976)**

("Severe Acute Respiratory Syndrome Coronavirus 2"[Supplementary Concept] OR "COVID-19"[Supplementary Concept] OR "Betacoronavirus"[MeSH Terms] OR "Coronavirus"[MeSH Terms] OR "covid*"[Text Word] OR "coronavirus*"[Text Word] OR "corona virus*"[Text Word] OR "ncov*"[Text Word] OR "n cov*"[Text Word] OR "sarscov*"[Text Word] OR "sars cov*"[Text Word] OR "2019ncov*"[Text Word] OR "2019 ncov*"[Text Word] OR "sars2*"[Text Word] OR "sars 2*"[Text Word])

**I**

**2 (816722)**

("Point-of-Care Testing"[MeSH Terms] OR "antigen"[Text Word] OR "lateral flow"[Text Word] OR "RDT"[Text Word] OR (("point of care*"[Text Word] OR "bedside*"[Text Word] OR "rapid*"[Text Word]) AND "test*"[Text Word]))

**3 (1637239)**

2019/12/01:2021/11/19[Date - Publication]

**1 AND 2 AND 3 (2990)**

## Web of Science Core Collection

P

| "covid*" OR  "coronavirus*" OR  "corona virus*" OR  "ncov*" OR  "n cov*" OR  "sarscov*" OR  "sars cov*" OR  "2019nCoV*" OR  "2019 nCoV*" OR  "sars2*" OR  "sars 2*" |
| --- |

I

| "antigen" OR  "Lateral flow" OR  "RDT" OR  (("Point of Care*" OR  "Bedside*" OR  "Rapid*")  AND  Test*)) |
| --- |

P

**1 (56968)**

TS=("covid*" OR "coronavirus*" OR "corona virus*" OR "ncov*" OR "n cov*" OR "sarscov*" OR "sars cov*" OR "2019nCoV*" OR "2019 nCoV*" OR "sars2*" OR "sars 2*")

I

**2 (34058)**

TS=("antigen" OR "Lateral flow" OR "RDT" OR (("Point of Care*" OR "Bedside*" OR "Rapid*")AND Test*))

**1 AND 2 (1568)**

Filter (year to date 2022/11/07)

## Bio_MedRxiv

https://europepmc.org/

P

| Covid* OR  Coronavirus* OR  "corona virus*" OR  Ncov* OR  "n cov*" OR  Sarscov* OR  "Sars cov*" OR  2019nCoV* OR  "2019 nCoV*" OR  sars2* OR  "sars 2*" |
| --- |

I

| "Antigen*" OR  "Lateral flow*" OR  "RDT" OR  "Rapid test*" OR  "Bedside*" OR  "Point of Care*" |
| --- |

**P AND I (2225)**

(covid* OR Coronavirus* OR "corona virus*" OR ncov* OR "n cov*" OR sarscov* OR "sars cov*" OR 2019nCov* OR "2019 nCov*" OR sars2* OR "sars 2*")

AND

(Antigen OR "Lateral flow" OR RDT OR "Rapid test*" OR Bedside* OR "Point of Care*")

AND

(PUBLISHER:MedRxiv OR PUBLISHER:BioRxiv)

AND

FIRST_PDATE:[2019-12-01 TO 2022-11-07]

# Supplement Text 2 – Supplementary Methods

**Assessment of methodological quality**

The instrument assesses four domains: research participant selection, index test, reference standard, and flow and timing. Each domain is assessed for bias using distinct signaling questions. In addition to assessing bias, the tool also examines the relevance of each study included in relation to the research topic across all domains. The evaluation of the individual studies can be found in the Supplementary file S2.

**Study selection and data extraction**

The selection of studies, extraction of data, evaluation of the quality of studies, and their independence from manufacturers were carried out as described previously (14). Two researchers conducted in-depth analyses of subcategories of an extended data set after the primary analysis.

The titles and abstracts of all potential publications were screened according to the concept of the study question by two reviewers (SK, KM). A full-text review was conducted on all publications considered potentially eligible by the selfsame. Any discrepancies were resolved by an additional reviewer (LEB, CMD).

Concerning the independence from manufacturers we examined whether a study received financial support from a test manufacturer (including the free provision of Ag-RDTs), whether any study author was affiliated with a test manufacturer, and if a respective conflict of interest was disclosed. If any of these aspects were present, studies were deemed not independent from the test maker; otherwise, they were regarded independent.

The complete extracted data items are available in the Supplemental file S2; a list of studies with a possible dependence and associated bias towards the manufacturer can be found in Supplementary File S3.

**Statistical analysis and data synthesis**

We obtained raw data from the studies using a standardized data extraction form and recalculated performance estimates using the collected. In an effort to collect as complete a picture as possible, we contacted the paper's authors in the conduct of the search for additional information regarding Ct-values, viral burden, and Ag-levels, if estimated. The aforementioned raw data can be found in the Supporting File S2.

When at least four data sets with at least 20 positive samples were available, we derived the estimates for sensitivity and specificity against RT_PCR and perform meta-analysis using a bivariate model (meta-analysis was implemented with “reitsma” command from the R package “mada,” version 0.5.11). Univariate random-effects inverse variance meta-analysis was done for pooled sensitivity analysis per Ct values performed (using the “metaprop” and “metagen” commands from the R package “meta,” version 5.5–0).  We predefined meta-analysis subgroups with these characteristics: Ct value range (<20, <25, <30, ≥20, ≥25, ≥30), IFU-conforming vs. non-IFU-conforming, patient age (<18 vs. ≥18 years), symptom presence (symptomatic vs. asymptomatic), and symptom duration (‘DoS ≤7 days’ vs. ‘DoS >7 days’). Some original studies presented the median Ct-value together with the first and third interquartile range (IQR) and/or minimum and maximum values instead of the sample mean and standard deviation. We used the quantile estimation approach to calculate the mean and standard deviation of the Ct-values for these studies.

Ct value thresholds were expanded by up to three points within each interval to accommodate heterogeneous data (e.g., Ct value range group <20 can include studies with Ct values ≤17 to ≤23). When categorizing by age, the age group under 18 years comprised samples from individuals aged <16 or <18, whereas the age group ≥18 years included samples from individuals aged ≥16 or ≥18. Furthermore, the sampling site was summarized as follows: nasopharyngeal (NP) alone or combined with other (e.g., oropharyngeal [OP]), OP alone, anterior nasal (AN) included AN and nasal mid-turbinate (NMT) samples. As, for the IFU conformity group, the compliance was judged based on the study team’s information provided in the publication. The symptom duration groups were divided as follows: the group with a duration of ≤7 days included durations of ≤4, ≤5, ≤6, 6 to 7, ≤7, and ≤9 days, while the group with a duration of >7 days included durations of >5, 6 to 10, 6 to 21, >7, and 8 to 14 days. Expanding the criteria for the Ct-value, age, and duration of symptoms subgroup led to some overlap within the groups. The web application CoVariants [19] was used to analyze each study's predominant Variants of Concern (VoCs during the indicated study period. The latest WHO classification was used to derive the specific VoCs.

We created forest plots to display the sensitivity and specificity of each test and assessed the heterogeneity between studies visually. Using Stata's “midas” command, version 15, we conducted the Deeks test for funnel-plot asymmetry, as advised to evaluate publication bias in diagnostic test accuracy meta-analyses. A slope coefficient p-value < 0.10 indicates substantial asymmetry. The remaining analyses were done with R 4.2.1 (R Foundation for Statistical Computing, Vienna, Austria).

Three sensitivity analyses were scheduled namely excluding case-control studies, preprints, and studies relying on manufacturers. We compared the sensitivity analysis results with the overall results to evaluate potential bias.

# Supplement Text 3 – Definitions and underlying Technologies

**Definitions**

For this work, we will follow the following definitions:

- Diagnostic test: “a test that is used to determine, verify or confirm a patient’s clinical condition as a sole determinant”(1)
- Point-of-care (POC) in vitro diagnostic (IVD) testing: “decentralised testing that is performed by a minimally trained healthcare professional near a patient and outside of central laboratory testing facilities and can result in an immediate decision for next steps of care” (2, 3)
- Instrument-based Ag RDTs: Ag RDTs that are instrument-depended, from large-scale atomised detection to small reading devices. Only those listed in WHO, European or US governmental healthcare documents and websites will be taken into consideration.

**Technologies**

The technologies underlying the investigated tests range from lateral flow immunochromatographic assays to upscaled Chemiluminescent enzyme immunoassays (CLEIA). The primary biochemical principle of every technology is that of an immunoassay using the selective binding interactions of antibody-antigen reactions to identify and quantify unknown analytes (protein, lipid, nucleic acid) (4). The main distinction is that most immunoassays use labels, such as those investigated here, while others don’t.

A further division is the two groups of heterogenous and homogenous immunoassays. A heterogeneous immunoassay, also named separation immunoassay, requires a multi-step analyte-antibody complex separation before analysis, while the homogenous does not. The labelled immunoassays can be further broken down into noncompetitive or competitive. The first ones use excess labelled antibodies to bind with the analyte making it more sensitive. In contrast, in the latter, the analyte competes with a specific quantity of labelled antigen for the antibody. Finally, the format is classified and described with the respective prefix by what antigen-antibody complexes are formed: direct, indirect and sandwich.

In our review, the investigated test belongs to the following subgroups divided by their reporter Chemiluminescence immunoassays (CLIA) and Fluorescent Immunoassays (FIA), where detection is done by fluorescent tracers or light-generating molecules and colourimetric Enzyme-linked immunosorbent assay (ELISA).

We further categorised them by being suitable for (near) point-of-care use or lab-based, depending on the principle, size of the reader and degree of automation.

POC and near-POC include two microfluidic immunofluorescence assays, (fluorescence) immunochromatography and one automated fluorescence immunoassay (mariPOC). The lab-based tests are a mixture of chemiluminescent enzyme immunoassays, chemiluminescent immunoassays, electrochemiluminescence immunoassays and semi-quantitative enzyme-linked immunosorbent assay.

In the following, the types of immunoassays will be explained:

ELISA is a colourimetric, chemiluminescent or fluorescent microwell plate-based assay used for the quantitation and detection of human proteins, immunoglobulins, antigens and other peptides through the binding between the target protein and a specific antibody that results in a detectable signal (5). This technique allows researchers to obtain precise and sensitive results in a relatively short time ranging from 1 to 5 h. To identify SARS-CoV-2 antigens, the principal of a sandwich ELISA is used to contain immobilised antibodies against SARS-CoV-2 antigens at the bottom of each well able to bind antigens contained in the serum samples of patients.

CLIAs, also known as chemiluminescent microparticle immunoassays, are automated assays that rely on mixing patient samples with magnetic, protein-coated microparticles and generate a light-based, luminescent readout. (6, 7)

The CLEIA method is a subgroup (to which the LumiraDx can be counted) in which antibodies are bound to magnetic particles, magnetised, and then reacted with a luminescent reagent to measure the amount of luminescence. (8)

For more facile and accurate detection of the assays, there are further techniques under investigation: New nanomaterials (9), molecules with an improved recognition capability – biorecognition elements like new type of antibodies or aptamers (10, 11) and devices suitable for the point-of-care bioassays combining the biorecognition elements with a sensor platform, biosensors(12), and similar point-of-care diagnostic means are progressively evolving in the COVID-19 diagnostics.

This immunological principle makes qualitative detection of an analyte and quantitative determination of its concentration possible. The measured optical signal is typically based on an accumulated sum of labels present in a probe region. Therefore, no precise information on single molecules or on the distribution and time trajectories of single molecules can be obtained.

1. WHO. The selection and use of essential in vitro diagnostics - TRS 1031 2021 [Available from: <https://www.who.int/publications/i/item/9789240019102>.

2. Pai NP, Vadnais C, Denkinger C, Engel N, Pai M. Point-of-Care Testing for Infectious Diseases: Diversity, Complexity, and Barriers in Low- And Middle-Income Countries. PLOS Medicine. 2012;9(9):e1001306.

3. World Health O. In vitro diagnostic medical devices (IVDs) used for the detection of high-risk human papillomavirus (HPV) genotypes in cervical cancer screening. Geneva: World Health Organization; 2018 2018.

4. Moser A, Carlson T. General principles of immunoassays. 2014. p. 6-19.

5. Alhajj M, Farhana A. Enzyme Linked Immunosorbent Assay. StatPearls. Treasure Island (FL)2022.

6. Mardian Y, Kosasih H, Karyana M, Neal A, Lau C-Y. Review of Current COVID-19 Diagnostics and Opportunities for Further Development. Front Med (Lausanne). 2021;8:615099-.

7. Chen D, Zhang Y, Xu Y, Shen T, Cheng G, Huang B, et al. Comparison of chemiluminescence immunoassay, enzyme-linked immunosorbent assay and passive agglutination for diagnosis of Mycoplasma pneumoniae infection. Ther Clin Risk Manag. 2018;14:1091-7.

8. Yamamoto K, Ohmagari N. Microbiological Testing for Coronavirus Disease 2019. JMA J. 2021;4(2):67-75.

9. Tharayil A, Rajakumari R, Kumar A, Choudhary MD, Palit P, Thomas S. New insights into application of nanoparticles in the diagnosis and screening of novel coronavirus (SARS-CoV-2). Emergent Materials. 2021;4(1):101-17.

10. Gupta R, Sagar P, Priyadarshi N, Kaul S, Sandhir R, Rishi V, et al. Nanotechnology-Based Approaches for the Detection of SARS-CoV-2. Frontiers in Nanotechnology. 2020;2.

11. Wu X, Chen Q, Li J, Liu Z. Diagnostic techniques for COVID-19: A mini-review. J Virol Methods. 2021;301:114437.

12. Seo G, Lee G, Kim MJ, Baek S-H, Choi M, Ku KB, et al. Rapid Detection of COVID-19 Causative Virus (SARS-CoV-2) in Human Nasopharyngeal Swab Specimens Using Field-Effect Transistor-Based Biosensor. ACS Nano. 2020;14(4):5135-42.
