# Supplementary material for: Clinical accuracy of instrument-based SARS-CoV-2 antigen diagnostic tests: a systematic review and meta-analysis
Source: Virol J. 2024 Apr 29;21:99. doi: 10.1186/s12985-024-02371-5 (PMC11059670; doi:10.1186/s12985-024-02371-5)
Supplement: Supplementary file 4 — Supplementary Material 4 [file 12985_2024_2371_MOESM4_ESM.docx]

**SUPPLEMENTARY MATERIAL**

File S1 – Prisma checklist, search terms, and definitions

File S2 – Raw data and data overview

File S3 – Studies potentially influenced by the test manufacturer

**SUPPLEMENTARY FIGURES AND TABLES**

**Figure S1 – Funnel Plot**


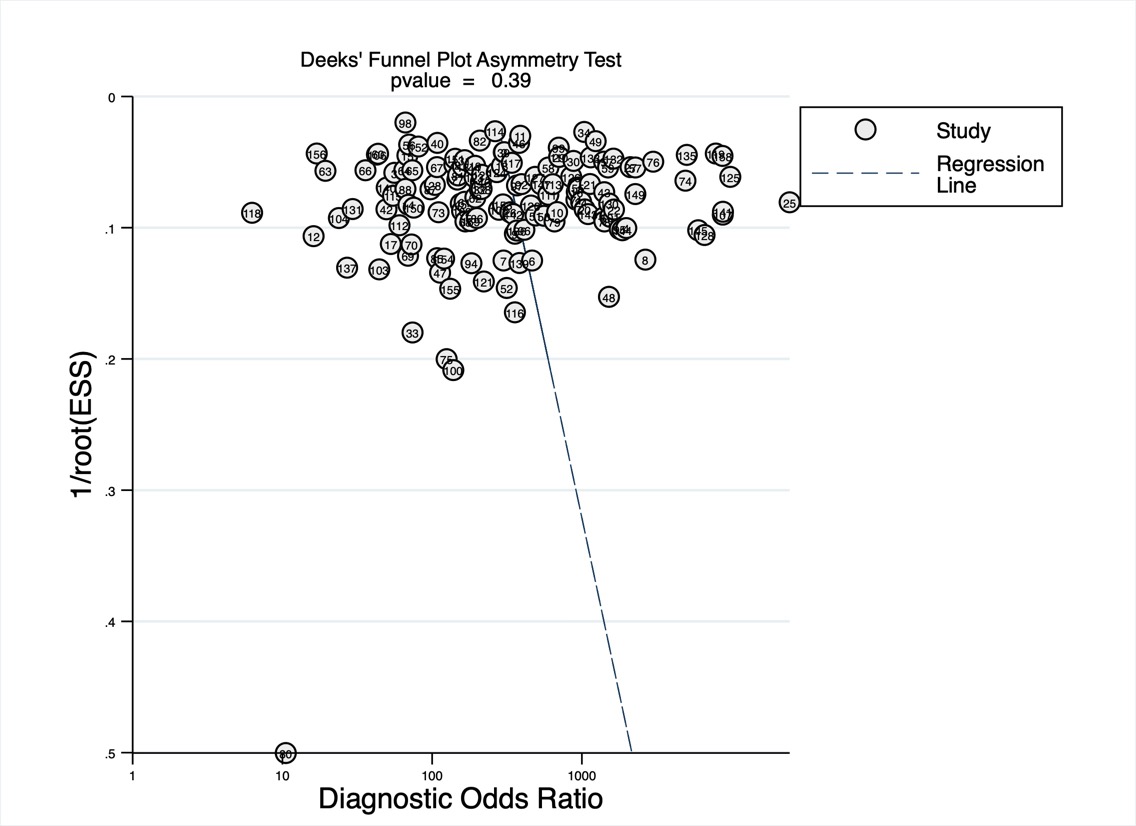


# Figure S2 – Forest plot of data included in bivariate analysis


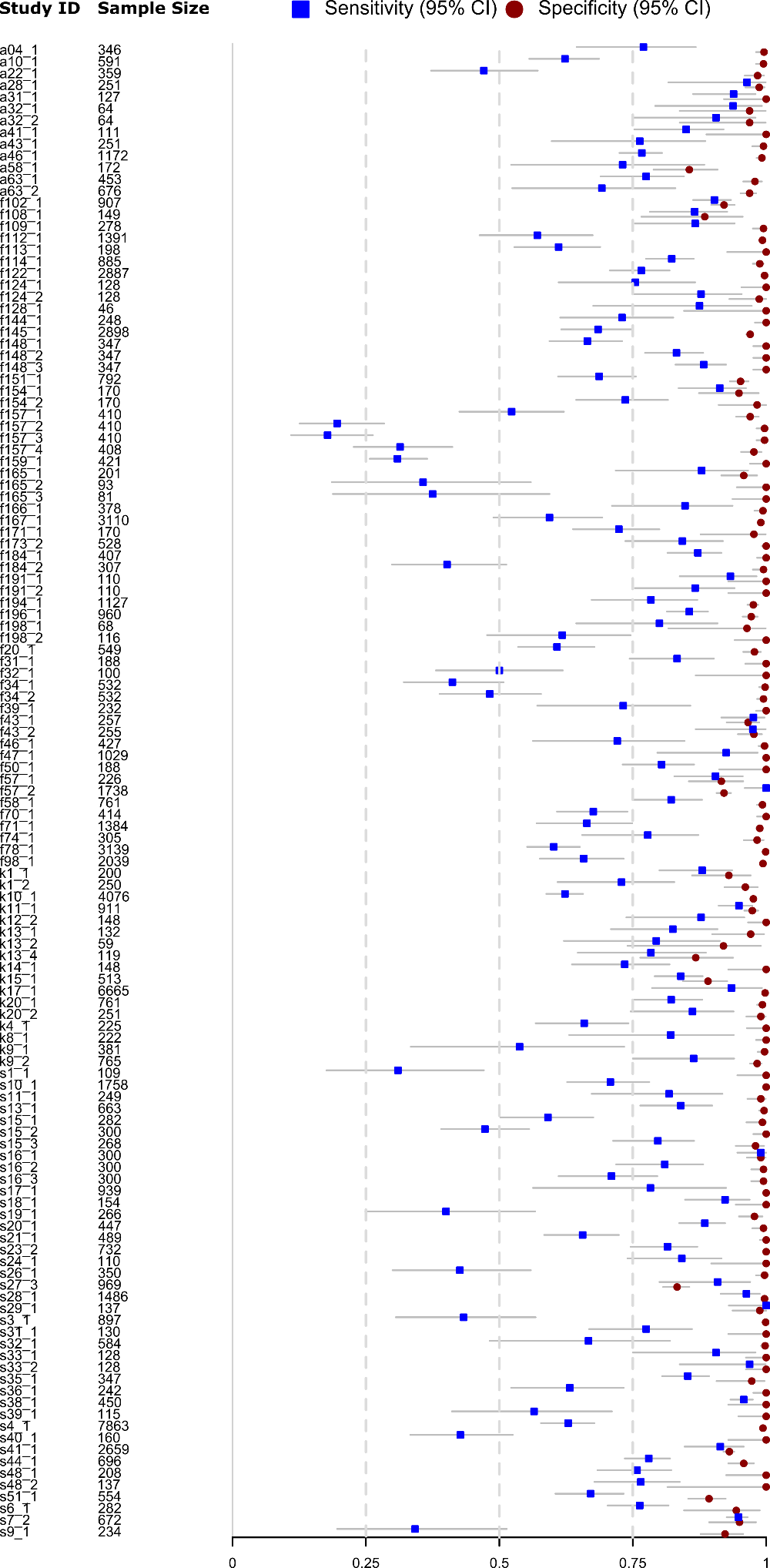


# Figure S3 – Forest plot of data included in univariate analysis - sensitivity


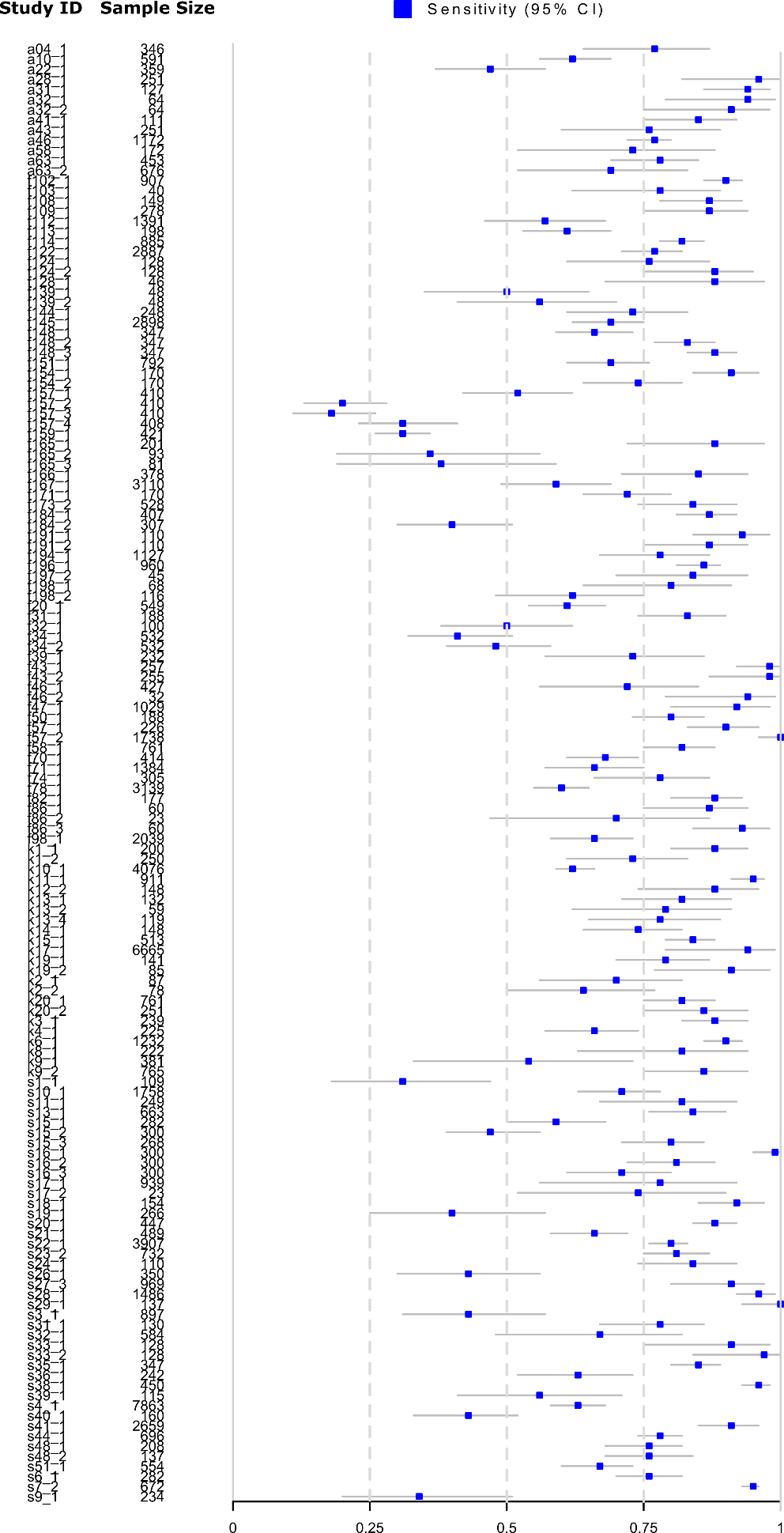


# Figure S4 – Forest plot of data included in univariate analysis - specificity


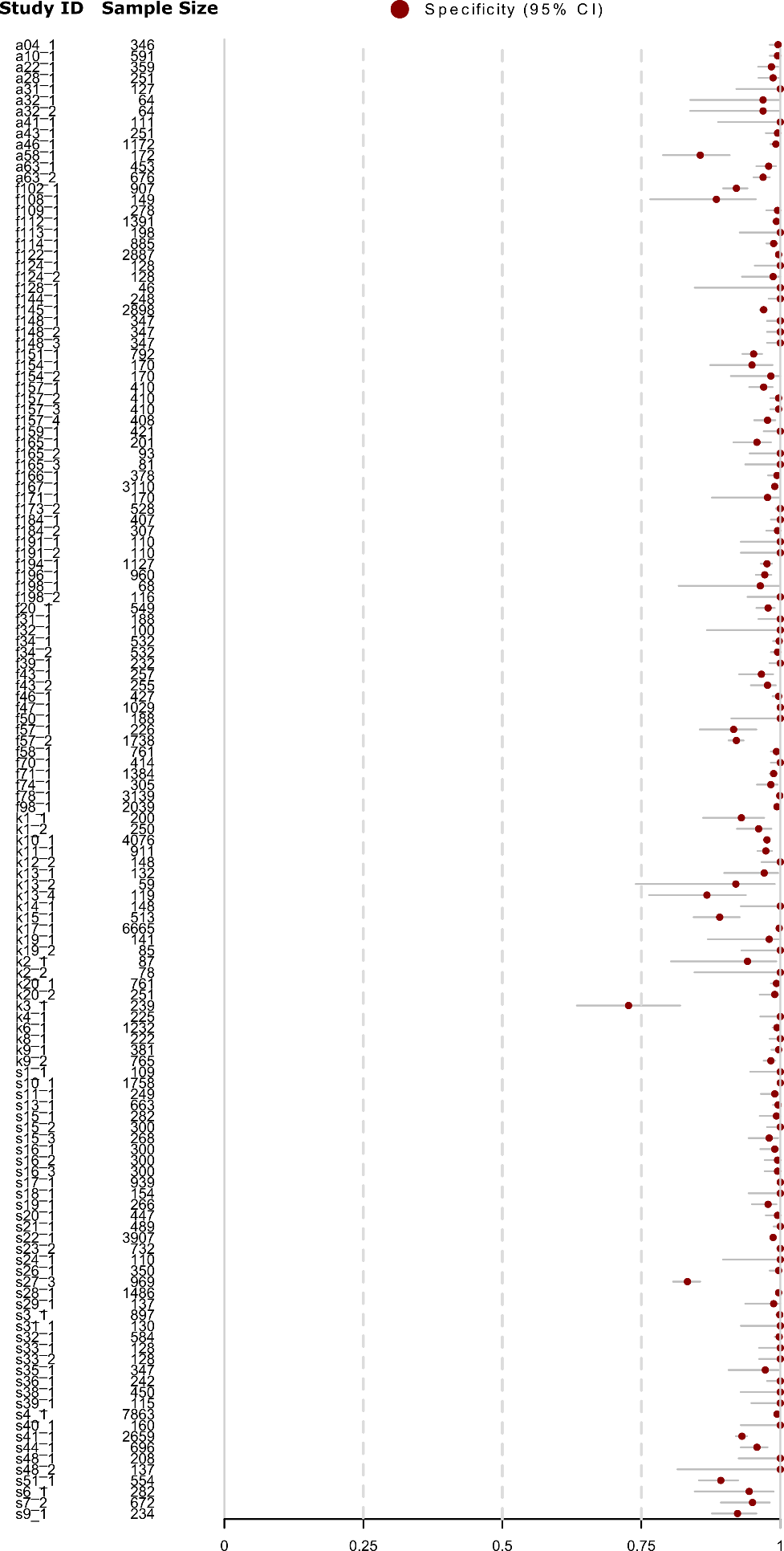


# Figure S5 – Pooled accuracy for the symptomatic and asymptomatic subgroups per intended setting

#
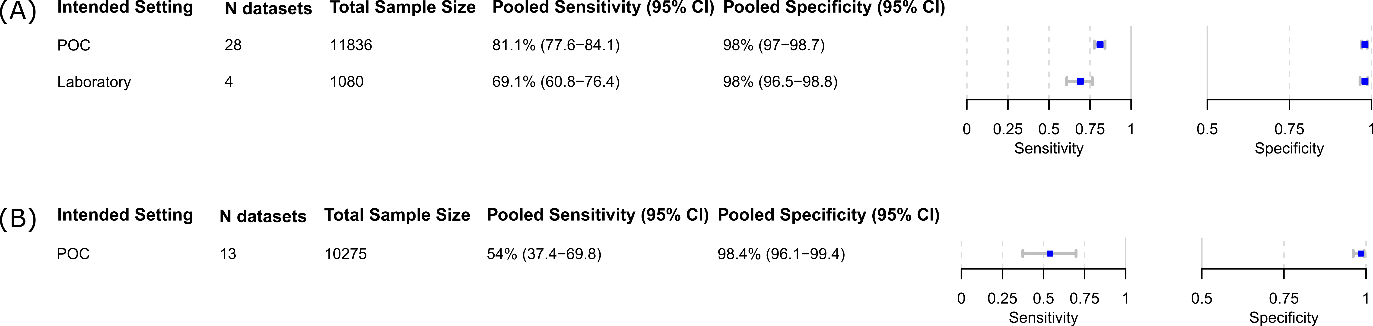


Figure S5 – Pooled accuracy for (A) the symptomatic subgroup and (B) the asymptomatic subgroup per intended use setting. Abbreviations: POC= point of care; CI = confidence interval

# Figure S6 – Sensitivity analysis excluding case-control studies


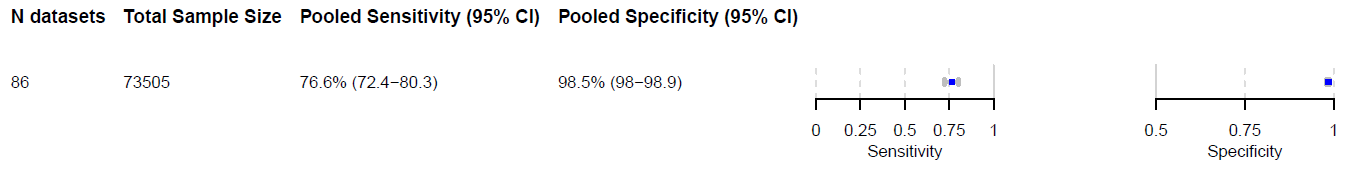


Figure S6 - Abbreviations: CI = confidence interval

# Figure S7 – Sensitivity analysis excluding preprints


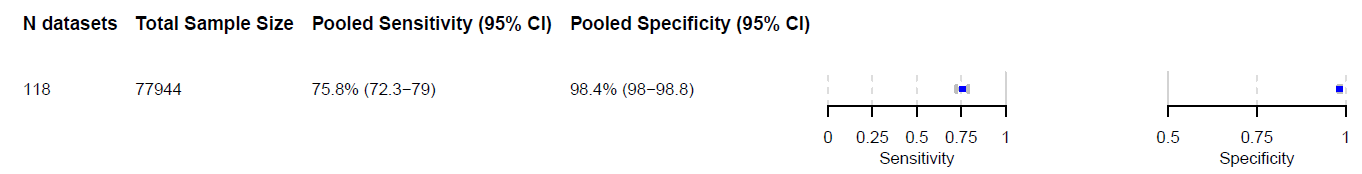


Figure S7 - Abbreviations: CI = confidence interval

# Figure S8 – Sensitivity analysis excluding manufacturer-dependent studies


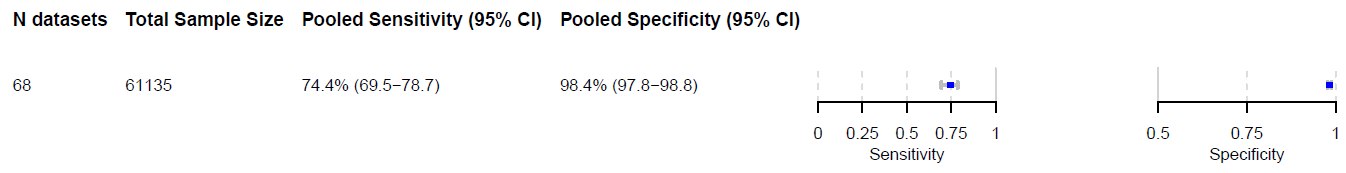


Figure S8 - Abbreviations: CI = confidence interval

# Figure S9 – Sensitivity analysis based on income level

High-income countries only:


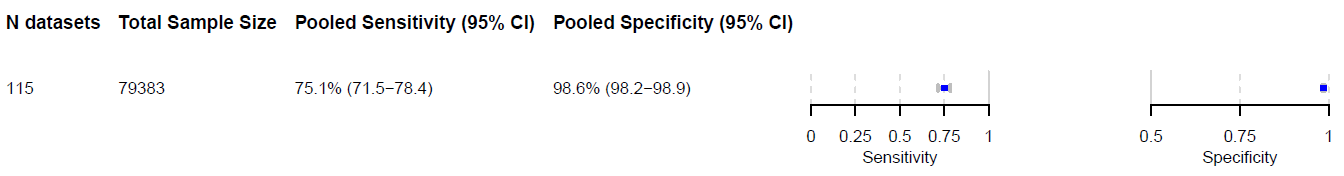


Figure S9A - Abbreviations: CI = confidence interval

Low- and middle-income countries only:
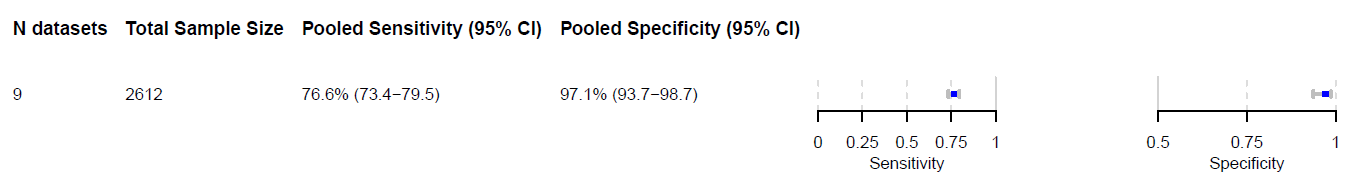


Figure S9B - Abbreviations: CI = confidence interval

# Table S1. Overview of iAg tests included in the review

| **Short Name** | **Company** | **Platform** | **Assay** | **Technology** | **Intended Use Setting** | **Time-to-Result** | **Number**  **of**  **Datasets** | **Number of Tests**  **Performed** | **References** |
| --- | --- | --- | --- | --- | --- | --- | --- | --- | --- |
| ADVIA Centaur | Siemens Healthineers | ADVIA Centaur (XP/XPT) XPT Immunoassay System | ADVIA Centaur SARS-CoV-2 Antigen Assay | CLIA | Lab-based | 23 minutes | 1 | 347 | (60) |
| AFIAS | Boditech Med | AFIAS-6 | AFIAS COVID-19 Ag | FIA | POC | 10-30 minutes | 2 | 846 | (70), (71) |
| Atellica | Siemens Healthineers | Atellica® Solution | Atellica IM SARS-CoV-2 Antigen Assay | CLIA | Lab-based | 26 minutes | 1 | 447 | (66) |
| BD Veritor | BD (Becton, Dickinson and Company) | BD Veritor^TM^ Plus Analyzer | BD Veritor™ System for Rapid Detection of SARS-CoV-2 | DIA | POC | 12-20 minutes | 17 | 11878 | (65), (72), (73), (74), (75), (76), (40), (77), (42), (44), (78), (52), (59), (24), (79), (80) |
| Bioeasy | Shenzhen Bioeasy Biotechnology | Immunofluorescence Analyzer EASY-11 | Bioeasy 2019-nCoV Ag Fluorescence Rapid Test Kit | FIA | POC | 10-20 minutes | 4 | 1137 | (34), (36), (81), (82) |
| Elecsys | Roche Diagnostics | cobas e 411, e 601, e 602, e 801 analyzers | Elecsys SARS-CoV-2 Antigen assay | ECLIA | Lab-based | 15-18 minutes | 12 | 5586 | (83), (84), (46), (85), (28), (86), (58), (87) |
| Euroimmun | Euroimmun Medizinische Labordiagnostika | Analyzer I-2P | SARS-CoV-2 Antigen ELISA | ELISA | Lab-based | n/a | 2 | 547 | (84), (88) |
| Exdia | Precision Biosensor | Exdia TRF analyzer | Exdia COVID-19 Ag | FIA | POC | 15-20 minutes | 2 | 832 | (73), (52) |
| FIAflex | ACON Laboratories | FIAflex™ Fluorescent Immunoassay Analyzer | FIAflex™ SARS-CoV-2 Antigen FIA | FIA | POC | 15 minutes | 1 | 347 | (44) |
| FREND | NanoEntek | FREND™ System | FREND^TM^ COVID-19 Ag test | FIA | POC | 3 minutes | 2 | 170 | (89), (90) |
| Gazelle | Hemex Health | Gazelle Reader | Gazelle COVID-19 | FIA | POC | n/a | 1 | 1486 | (91) |
| HISCL | Sysmex Corporation | HISCL®‐5000 / HISCL®‐800 | HISCL® SARS‐CoV‐2 Ag Assay kit | CLEIA | Lab-based | 17 minutes | 3 | 1625 | (92), (64) |
| ichroma | Boditech Med | ichroma™ | ichroma™ COVID-19 Ag Test | FIA | POC | 15-30 minutes | 1 | 232 | (93) |
| LIAISON | DiaSorin S.p.A. | LIAISON® XL | LIAISON® SARS-CoV-2 Ag | CLIA | Lab-based | 45 minutes | 10 | 4087 | (39), (43), (84), (94), (95), (96), (57), (61), (62) |
| Lumipulse G | Fujirebio | Lumipulse® G1200 | Lumipulse® G SARS-CoV-2 Ag | CLEIA | Lab-based | 30-60 minutes | 16 | 8953 | (97), (38), (98), (99), (84), (70), (48), (50), (100), (101), (53), (102), (103) |
| LumiraDx | LumiraDx | LumiraDx™ Platform | LumiraDx SARS-CoV-2 Ag test | FIA | POC | 12-15 minutes | 24 | 10136 | (104), (31), (105), (106), (89), (107), (108), (41), (109), (110), (44), (111), (112), (113), (114), (115), (26), (116), (117), (52), (55), (25) |
| mariPOC | ArcDia International | mariPOC® test system | mariPOC SARS-CoV-2 test | FIA | POC | 20-55 minutes | 4 | 1020 | (47), (118) |
| PCL | PCL | n/a | PCL COVID-19 Ag Rapid FIA test | FIA | POC | <30 minutes | 2 | 353 | (119), (54) |
| RapidTesta | Sekisui Medical | n/a | RapidTesta SARS-CoV-2 | DIA | POC | 10 minutes | 1 | 1127 | (120) |
| MSD S-PLEX | MesoScale Diagnostics | MESO SECTOR S 600 Reader | MSD S-PLEX SARS-CoV-2 N assay | ECLIA | Lab-based | 4-5 hours | 5 | 846 | (121), (49), (13) |
| Sofia | Quidel | Sofia Fluorescent Immunoassay Analyzer | Sofia SARS Antigen FIA | FIA | POC | 15 minutes | 22 | 20970 | (122), (35), (123), (82), (37), (124), (125), (126), (127), (42), (121), (119), (128), (129), (130), (131), (51) |
| STANDARD F | SD Biosensor | STANDARD^TM^ F2400 Analyzer / STANDARD^TM^ F2400 Analyzer | STANDARD^TM^ F COVID-19 Ag FIA | FIA | POC | 10-30 minutes | 18 | 19617 | (132), (133), (35), (30), (134), (89), (135), (70), (136), (90), (137), (71), (138), (51), (56), (63), (139) |
| VITROS | Ortho Clinical Diagnostics | VITROS® 3600 Immunodiagnostic System | VITROS Immunodiagnostic Products SARS-CoV-2 Antigen test | CLIA | Lab-based | 48 minutes | 6 | 2774 | (140), (99), (45), (27), (141) |
| Wantai | Beijing Wantai Biological Pharmacy Enterprise | n/a | Wantai SARS-CoV-2 Antigen Rapid Test (FIA) | FIA | POC | 20 minutes | 2 | 718 | (51), (142) |

Table S1 - Overview of iAg tests included in the review. Abbreviations: CLIA = Chemiluminescence immunoassay, FIA = Fluorescence immunoassay, DIA = Diffusion immunoassay, CLEIA = Chemiluminescence enzyme immunoassay, ECLIA = Electrochemiluminescence immunoassay, ELISA = Enzyme-linked immunosorbent assay, POC = Point-of-care
